# M1 macrophage‐derived exosomes promote autoimmune liver injury by transferring long noncoding RNA H19 to hepatocytes

**DOI:** 10.1002/mco2.303

**Published:** 2023-06-29

**Authors:** Yongting Zhang, Liang Hong, Xuehui Li, Yuyu Li, Xujun Zhang, Jingjing Jiang, Fan Shi, Hongyan Diao

**Affiliations:** ^1^ State Key Laboratory for Diagnosis & Treatment of Infectious Diseases National Clinical Research Center for Infectious Disease Collaborative Innovation Center for Diagnosis & Treatment of Infectious Diseases The First Affiliated Hospital College of Medicine Zhejiang University Hangzhou China

**Keywords:** apoptosis, exosomes, H19, liver injury, macrophage

## Abstract

Exosomes mediate intercellular communication by transmitting active molecules. The function of long noncoding RNA (lncRNA) H19 in autoimmune liver injury is unclear. Concanavalin A (ConA)‐induced liver injury is well‐characterized immune‐mediated hepatitis. Here, we showed that lncRNA H19 expression was increased in the liver after ConA treatment, accompanied by increased exosome secretion. Moreover, injection of AAV‐H19 aggravated ConA‐induced hepatitis, with an increase in hepatocyte apoptosis. However, GW4869, an exosome inhibitor, alleviated ConA‐induced liver injury and inhibited the upregulation of lncRNA H19. Intriguingly, lncRNA H19 expression in the liver was significantly downregulated, after macrophage depletion. Importantly, the lncRNA H19 was primarily expressed in type I macrophage (M1) and encapsulated in M1‐derived exosomes. Furthermore, H19 was transported from M1 to hepatocytes via exosomes, and exosomal H19 dramatically induced hepatocytes apoptosis both in vitro and vivo. Mechanistically, H19 upregulated the transcription of hypoxia‐inducible factor‐1 alpha (HIF‐1α), which accumulated in the cytoplasm and mediated hepatocyte apoptosis by upregulating p53. M1‐derived exosomal lncRNA H19 plays a pivotal role in ConA‐induced hepatitis through the HIF‐1α–p53 signaling pathway. These findings identify M1 macrophage‐derived exosomal H19 as a novel target for the treatment of autoimmune liver diseases.

## Graphical abstract

**Scheme depicting M1‐derived exosomes transfer of lncRNA H19 into hepatocytes and its role in the progression of conA‐induced hepatitis**. In conA‐induced hepatitis, the lncRNA H19 is primarily expressed in type I macrophage (M1). M1 releases exosomes, which carry H19 to hepatocytes. In hepatocyte, H19 upregulates the transcription of HIF1α, which accumulates in the cytoplasm, and subsequently upregulates the protein level of P53, thereby leading to hepatocyte apoptosis, and liver injury.

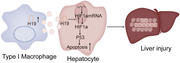

## 1 INTRODUCTION

Autoimmune hepatitis (AIH) is a chronic immune cell‐mediated liver disease with unknown cause.^1^, ^2^ Despite the fact that hepatocyte apoptosis played a key role in AIH progression, the precise mechanisms of cell death regulation remained poorly understood. Administration of concanavalin A (ConA) has been widely recognized as an appropriate model of immune‐mediated liver injury in mice. ConA‐induced liver injury is characterized as excessive activation of T cells, natural killer T (NKT) cells, macrophages, and cytokine release, such as tumor necrosis factor‐α (TNF‐α), interferon‐gamma (IFN‐γ), and interleukin‐6 (IL‐6), which lead to hepatocyte apoptosis.^3^, ^4^ Recent studies have reported that depletion of macrophage by clodronate liposomes or gadolinium chloride alleviated ConA‐induced liver injury but did not inhibit cytokine release.^5^, ^6^ Our previous study showed that dampening macrophage necroptosis can alleviate ConA‐induced liver injury by blocking TNF receptor (TNFR)‐1.^7^ However, the underlying pathophysiological mechanisms of macrophages remain incompletely understood.

Exosomes are ranged from 40 to 160 nm in diameter derived from the endosome, via transferring biological cargo, including lipids, proteins, and ncRNAs.^8^ Exosomes, derived from various cell types, can be taken up by endocytosis and the cargo contained within them was transferred into the recipient cells, which regulate the function of target cells, especially in liver pathology.^9^ Hou reported that after IL‐6 treatment, the production of miR‐223‐enriched exosomes derived from myeloid cells in NAFLD was elevated, and the exosomal miR‐223 was transferred into hepatocytes to reduce profibrotic genes.^10^ In addition, exosomal miR‐106b‐5p derived from Trem2‐deficient macrophages impaired hepatocytic mitochondrial structure and energy supply, which exacerbated the progression of NAFLD and increased susceptibility to sepsis.^11^, ^12^, ^13^ However, the role of exosomes in ConA‐induced liver injury remains unclear.

Long noncoding RNA (lncRNA) H19, an imprinted gene, is highly expressed in the embryonic liver but repressed significantly after birth.^14^ Interestingly, lncRNA H19 was significantly increased in hepatocytes in nonalcoholic steatohepatitis (NASH), cholestatic, and carbon tetrachloride (CCl4)‐induced liver fibrosis.^14^, ^15^, ^16^ Moreover, exosomal H19 accumulated in hepatic stellate cells (HSCs) and induced HSC activation, leading to liver fibrosis in bile duct ligation models.^16^, ^17^ However, the role of H19 in ConA‐induced hepatitis remains largely undefined.

Herein, we aimed to investigate whether H19 and exosomes play a key role in ConA‐induced hepatitis. In our studies, we found that the levels of H19 and exosomes in the liver were increased, after ConA treatment. GW4869, an exosome inhibitor, inhibited the upregulation of H19. Moreover, H19 was mainly expressed in M1. More importantly, we found that H19 was transported from M1 to hepatocytes via exosomes and activated the hypoxia‐inducible factor‐1 alpha (HIF‐1α)‐ p53 signaling pathway, consequently leading to hepatocyte apoptosis. Our findings shed new light on the role of macrophages in AIH, suggesting M1‐derived exosomal H19 is involved in AIH and may be a novel target for AIH.

## 2 RESULTS

### 2.1 Both lncRNA H19 and exosomes were elevated in ConA‐induced hepatitis

**FIGURE 1:**
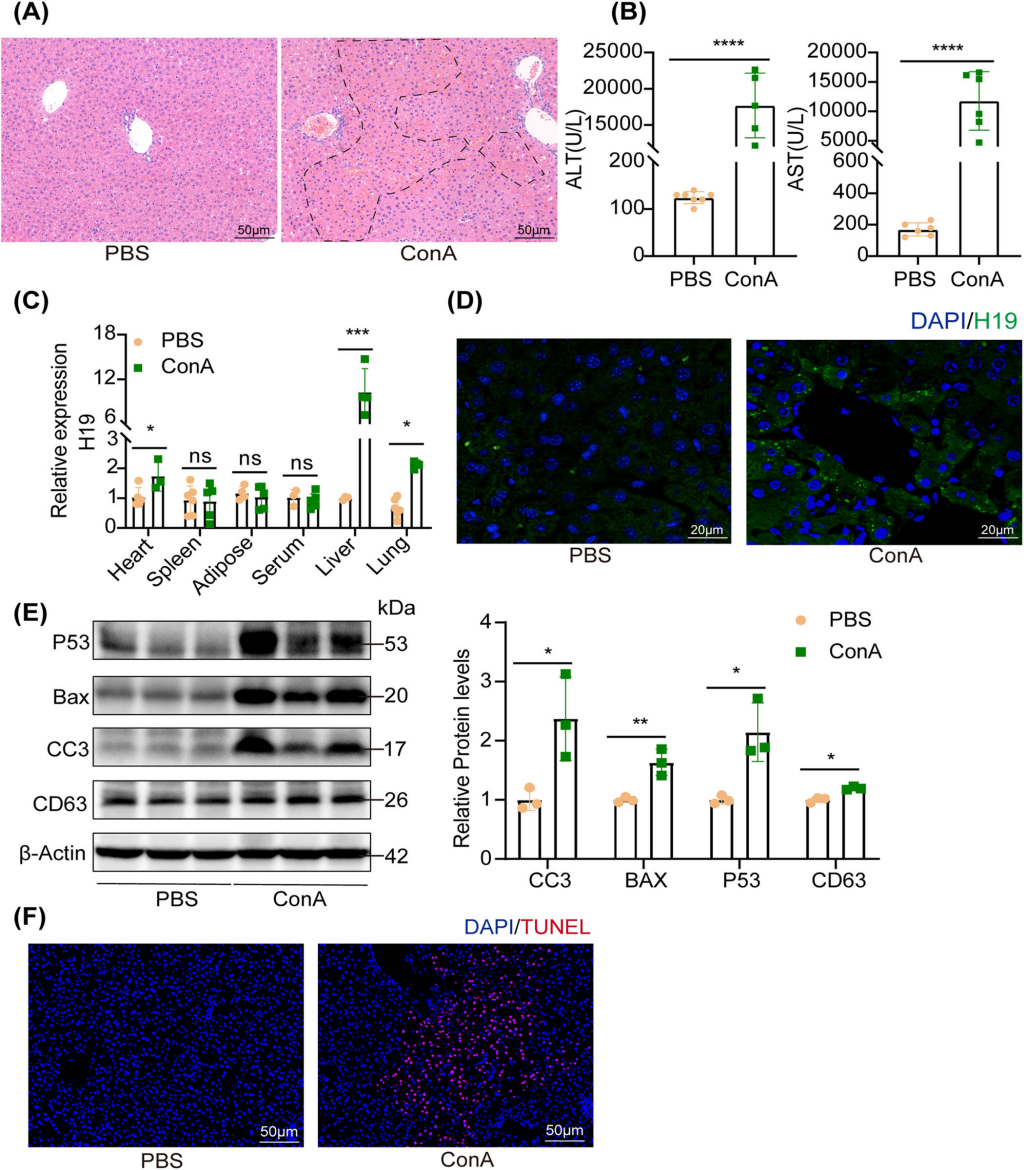
H19 expression and exosome production increased in ConA‐induced hepatitis. (A) Representative H&E staining of the liver tissue sections, scale bar = 50 μm (dashed line showed necrotic area). (B) The serum levels of ALT and AST after ConA injection, (*n* = 5–7). (C) H19 mRNA levels in different tissues were analyzed by RT‐PCR post‐ConA injection, (*n* = 4–6). (D) Representative images of FISH targeting H19 in the liver (green), scale bar = 20 μm. (E) Western blot showed the expression of CD63 and apoptotic proteins, including cleaved caspase‐3 (CC3), BAX, and p53, in the liver of mice subjected to ConA injection. (F) Representative images of TUNEL staining showed hepatocyte apoptosis in ConA‐treated mice, scale bar = 50 μm. Statistical analysis showed the mean ± SD; ns: not statistically significant, *p < 0.05, **p < 0.01, ***p < 0.001.

Previous studies demonstrated that H19 was highly elevated in the liver of NASH, cholestatic, and CCL4‐induced liver fibrosis.^14^, ^15^, ^16^ Here, we found that H19 was also significantly elevated in the liver and not in other organs of ConA‐treated mice (Figures 1A–C and S1A). We also performed H19 in situ hybridization. As shown in Figure 1D, H19 was indeed localized in the hepatocytes. Intriguingly, H19 was highly expressed in the embryonic liver, but at a low level in the adult liver. The increased H19 in the liver can be attributed to exosomes, which transport biological cargoes, including lipids, proteins, and noncoding RNAs. CD63 regulated exosome secretion and was detected by western blot. We found a significantly increased level of CD63 in ConA‐treated mice (Figure 1E). Moreover, the H19 and exosomes were also elevated in AIH patients (Figure S1C). In addition, the number of TUNEL‐positive cells and apoptotic proteins also increased (Figures 1E and F and S1B). These results indicated H19 in the liver was significantly increased, accompanied by an increased level of exosomes, after ConA treatment.

### 2.2 Liver‐specific overexpression of H19 aggravated ConA‐induced hepatitis

**FIGURE 2:**
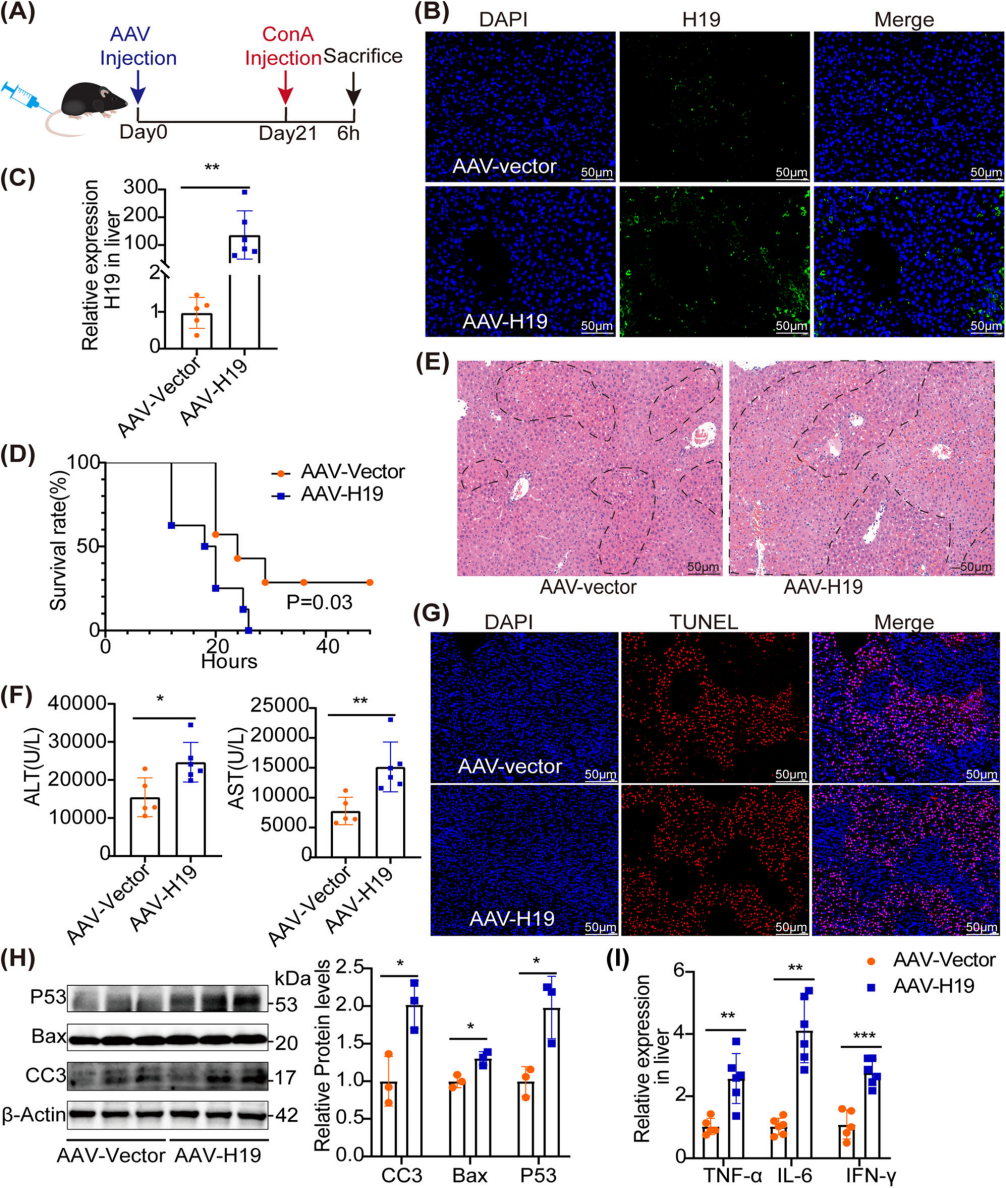
Overexpressed H19 aggravated ConA‐induced hepatitis. (A) Mice were injected with AAV via the tail vein before ConA treatment and were sacrificed. (B) Representative images of FISH of H19 expression in the liver after injection with AAV‐H19, scale bar = 20 μm (dashed line showed necrotic area). (C) RT‐PCR showing the expression of H19 in the liver from ConA mice treated with AAV‐H19 (*n* = 5–6). (D) Survival rates of AAV‐H19 mice and AAV‐Vector controls after a lethal dose of ConA treatment (ConA: 40 μ g/g) (*p < 0.05) (*n* = 9). (E) Representative images showing hepatic injury (scale bar = 50 μm) and (F) The serum levels of ALT and AST in ConA‐treated mice injected with either AAV‐Vector or AAV‐H19 (*n* = 5–6). (G) Representative images of TUNEL staining showing hepatocytes apoptosis in the liver, scale bar = 50 μm. (H) Western blot showed the expression of apoptotic proteins in the liver. (I) RT‐PCR showed the mRNA levels of inflammatory markers, including IL‐6, TNF‐α, and IFN‐γ (*n* = 5–6). Statistical analysis showed the mean ± SD; ns: not statistically significant, *p < 0.05, **p < 0.01, ***p < 0.001.

To further evaluate the function of hepatic H19, we generated an AAV8‐harboring empty vector (called AAV8‐Vector) and an H19 full‐length vector (called AAV8‐H19). They were injected into the tail vein respectively, 3 weeks before ConA treatment (Figure 2A). The hepatic expression of H19 in mice delivered with AAV8‐H19 was significantly increased, as compared with controls treated with the AAV8‐Vector (Figures 2B and C). When the mice were treated with a lethal dose of ConA, the AAV‐H19 mice were found to be more susceptible to ConA‐induced hepatitis, as they displayed a greater mortality rate than the AAV‐Vector controls (Figure 2D). The above result was attributed to more severe liver injury, with higher Ishak scores (Figures 2E and S2A). Consistently, the liver of H19‐overexpressed showed a significantly increased level of hepatic apoptosis, including TUNEL staining (Figures 2G and S2B), p53, BAX, and cleaved caspase‐3 (CC3), post‐ConA injection (Figure 2H). The mRNA levels of the inflammatory cytokines were also elevated (Figure 2I). Furthermore, hepatic H19‐overexpressed in control mice did not induce significant liver injury (Figure S9). Conversely, the knockdown of hepatic H19 attenuated ConA‐induced liver injury (Figure S10). Collectively, these results indicated that increased H19 exacerbated ConA‐induced hepatitis.

### 2.3 Exosomes inhibitor GW4869 attenuated ConA‐induced hepatitis

**FIGURE 3:**
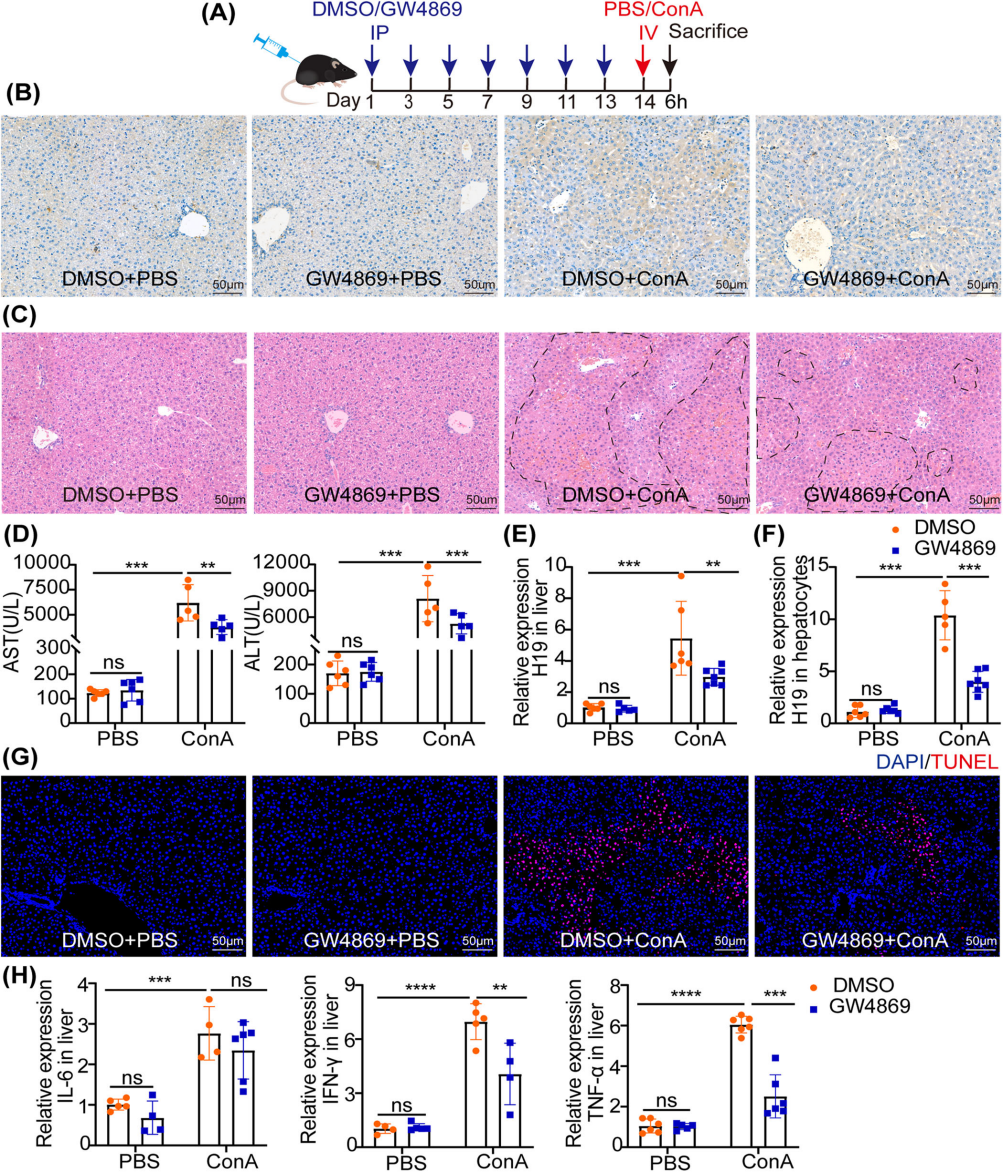
The exosome inhibitor GW4869 attenuated ConA‐induced hepatitis. (A) Schematic illustration of the experimental procedure analyzing the effects of GW4869 on ConA‐induced hepatic injury. (B) Representative immunohistochemistry staining of CD63, scale bar = 50 μm (dashed line showed necrotic area). (C) Representative images of histologic liver injury (scale bar = 50 μm). (D) The serum levels of ALT and AST, after ConA injection with or without GW4869 (*n* = 5–7). (E and F) RT‐PCR analysis of H19 levels of the liver and hepatocytes, from mice subjected to ConA with or without GW4869 (*n* = 5–7). (G) Representative images of TUNEL staining, scale bar = 50 μm. (H) RT‐PCR showed the mRNA levels of the inflammatory markers, including IL‐6, TNF‐α, and IFN‐γ (*n* = 5–7). Statistical analysis showed the mean ± SD; ns: not statistically significant, *p < 0.05, **p < 0.01, ***p < 0.001.

Next, the exosome secretion inhibitor, GW4869, was used to investigate the role of exosomes in ConA‐induced hepatic injury. After pretreatment with GW4869 by intraperitoneal injection (Figure 3A), the level of CD63 significantly decreased, which indicated that exosomes were depleted successfully in the liver (Figures 3B and S3A). Meanwhile, administration of GW4869 significantly improved ConA‐induced liver injury (Figures 3C and D). Treatment with GW4869 decreased the upregulation of H19 in the ConA‐treated liver (Figure 3E), as well as in hepatocytes (Figure 3F), which were comparable to the control group. It suggested that the upregulation of H19 in hepatocytes was attributed to exosomes, with unknown cell sources. Moreover, GW4869 treatment reduced TUNEL‐positive numbers (Figures 3G and S3B), and the levels of inflammatory cytokines (Figure 3H), including IL‐6, TNF‐α, and IFN‐γ, in the liver of ConA‐treated mice. The inhibition of exosomes with GW4869 alleviated ConA‐induced hepatitis and decreased the expression of H19.

### 2.4 H19 was primarily expressed in type I macrophage

**FIGURE 4:**
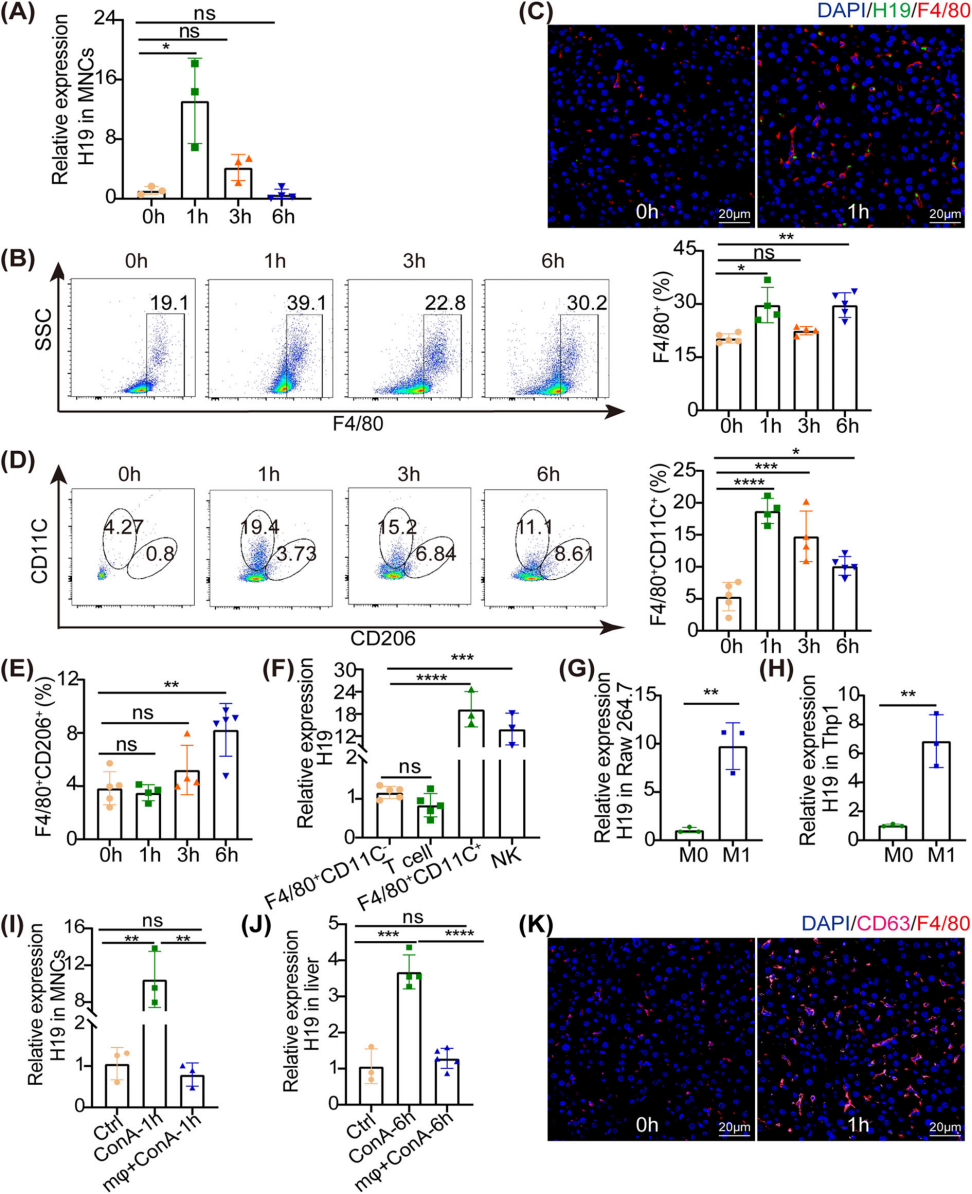
H19 was primarily expressed in M1. (A) RT‐PCR showed fold changes in H19 of MNCs isolated from the liver of ConA‐treated mice (*n* = 3–4). (B) Flow cytometry analysis of F4/80^+^ in MNCs from ConA‐treated mice (*n* = 4–5). (C) Colocalization of F4/80 (red) and H19 (green) in the liver 1 h post‐ConA treatment, scale bar = 20 μm. (D and E) Flow cytometry analysis of F4/80^+^CD11C^+^ and F4/80^+^CD206^+^ in mononuclear cells (MNCs) isolated from ConA‐treated mice (*n* = 4–5). (F) RT‐PCR analysis of H19 in NK cells, T cells, and macrophages from the injured liver (*n* = 3–5). (G and H) The expression of H19 was determined by RT‐PCR after RAW264.7 and THP‐1 were simultaneously stimulated with 20 ng/mL IFN‐γ and100 ng/mL LPS for 24 h. (I) The H19 level in the MNCs isolated from the liver 1 h post‐ConA treatment with or without clodronate liposome was detected by RT‐PCR (*n* = 3). (J) The level of hepatic H19 from mice 6 h post‐ConA treatment with or without clodronate liposome was determined by RT‐PCR (*n* = 3–5). (K) Colocalization of F4/80 (red) and CD63 (pink) in the liver 1 h post‐ConA treatment, scale bar = 20 μm. Statistical analysis showed the mean ± SD; ns: not statistically significant, *p < 0.05, **p < 0.01, ***p < 0.001.

The above data suggested that the upregulation of H19 in hepatocytes was attributed to exosome transfer. Previous studies demonstrated lncRNAs were also expressed in immune cells. ConA‐induced hepatitis was characterized as immune cell‐mediated liver injury, including T cell, NKT cells, and macrophage.^3^, ^18^ It remains to be investigated whether or not H19 is expressed in immune cells. We isolated the mononuclear cells (MNCs) from the liver and verified H19 was highly expressed at 1 h (Figure 4A). The flow cytometry showed an increased percentage of F4/80^+^ cells (Figure 4B), whereas no significant change was in NK cells or NKT cells at 1 h (Figures S4A–C). We then performed H19 in situ hybridization and costained the macrophage‐specific marker F4/80. As shown in Figure 4C, H19 was indeed localized in macrophages.

Macrophages were typically categorized into the proinflammatory “type I macrophage (M1)” and anti‐inflammatory “type II macrophage.” We found the percentage of CD11c^+^F4/80^+^ was increased in 1 h after ConA injection, not CD206^+^F4/80^+^ (Figures 4D and E). Additionally, we isolated CD11c^+^F4/80^+^, T cells, and NK cells from the liver of ConA‐treated mice, and found that H19 was mainly expressed in CD11c^+^F4/80^+^, with a relatively low expression level in T cells, NK cells, and CD11c^−^F4/80^+^ (Figure 4F). In vitro, H19 was also amplified in mouse RAW246.7 macrophages or human THP‐1, under either LPS plus IFN‐γ stimulus (Figures 4G–H), which differentiated M1 (Figures S4D–G). To further detect whether the upregulation of hepatic H19 was derived from M1 during ConA treatment, clodronate liposome was administered to deplete macrophages (Figures S5A and B). Clodronate liposome treatment alleviated ConA‐induced liver injury^5^ (Figures S5C and D). Meanwhile, TUNEL staining and proapoptotic markers were both significantly reduced to almost the level of control mice (Figures S5E–G); a decrease in the levels of inflammatory cytokines was also observed (Figure S5H). Moreover, the expression of H19 in MNCs was downregulated after macrophage depletion (Figure 4I), and the depletion of macrophages was paralleled by the downregulation of H19 in the liver (Figure 4J). It suggested that H19 in the liver originated, at least in part, from macrophages. Surprisingly, we found the exosome secretion, CD63, was significantly increased in macrophages (Figure 4K). All the above findings suggested H19 was primarily expressed in M1 and was transferred into hepatocytes.

### 2.5 M1 secreted exosomal H19

**FIGURE 5:**
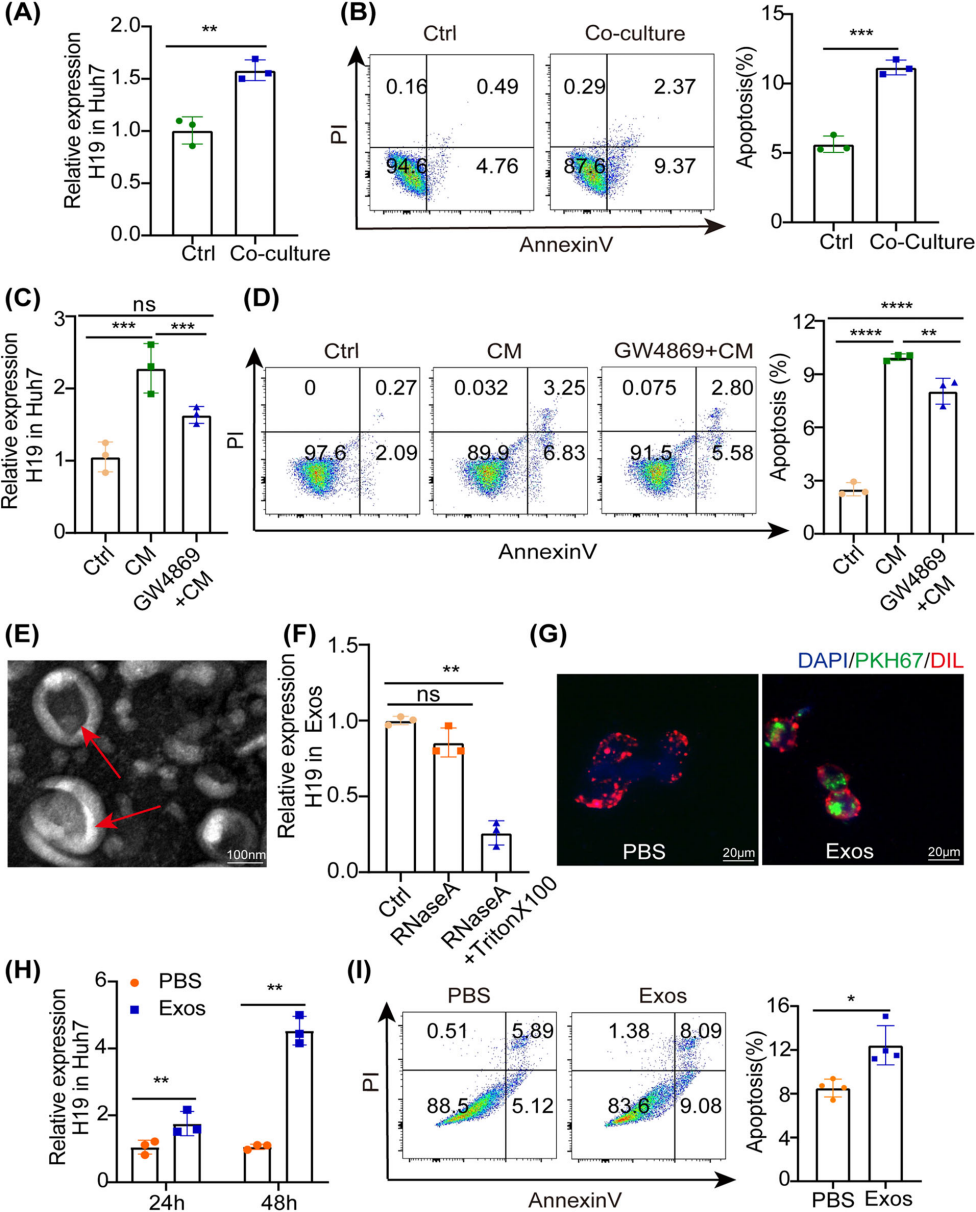
M1 secreted exosomal H19. (A) THP‐1 was stimulated by LPS/IFN‐γ simultaneously and cocultured with Huh7 cells in a trans‐well plate (0.4 μm). Then, the level of H19 in Huh7 was detected by RT‐PCR. (B) Apoptosis in Huh7 cells was detected by Annexin V‐PI, after coculture. (C) RT‐PCR analysis of H19 and (D) Flow cytometry analysis of apoptosis in Huh7 cells incubated with cell medium (CM) or GW4869 + CM, which was collected from M1 pretreated with exosome inhibitor (GW4869, 10 μM). (E) Representative transmission electron microscopy (TEM) image showing a cup‐shaped vesicle, arrows marked cup‐shaped vesicle with exosome, scale bar = 100 nm. (F) RT‐PCR analysis of H19 expression in M1‐secreted exosomes (Exos) treated with RNase A alone or in combination with Triton X‐100. (G) Confocal fluorescence microscopy images of Huh7 cells incubated with PKH67‐labeled M1‐Exos (green), scale bar = 20 μm. (H) The expression of H19 in Huh7 was detected by RT‐PCR. (I) Analysis of apoptosis in Huh7 cells after treatment with Exos or PBS by Annexin V‐PI. Statistical analysis showed the mean ± SD; ns: not statistically significant, *p < 0.05, **p < 0.01, ***p < 0.001.

To investigate whether the upregulation of H19 in hepatocytes was attributed to the exosomes derived from M1, M1 was cocultured in a 0.4 μm transwell system for 24 h, in which soluble factors were exchanged freely, but cell membranes were impermeable. We found a minor increase of H19 was detected in Huh7 (Figure 5A). In addition, the apoptosis in Huh7 was increased after coculture (Figure 5B). Furthermore, coculture was performed with a conditioned medium (CM) derived from M1 or CM pretreated with an exosome inhibitor (GW4869) for 24 h. GW4869 inhibited the upregulation of H19 (Figure 5C) and decreased the ratio of apoptosis in Huh7 (Figure 5D). It prompted us to investigate whether the upregulation of H19 in Huh7 was due to exosome transfer.

Exosomes were then isolated from the M1‐derived CM by standard differential ultracentrifugation (Figure S6A). Transmission electron microscopy (TEM) showed a cup‐shaped vesicle of about 100 nm in diameter (Figure 5E). Western blot analysis revealed that these exosomes were positive for TSG101, Alix, and CD63 (Figure S6B). Most importantly, H19 expression in exosomes was unchanged upon treatment with RNase alone but decreased significantly following simultaneous treatment with RNase A and Triton X‐100 (Figure 5F). Then, we tested whether these M1‐derived exosomes can be taken up by hepatocytes. These exosomes were labeled with PKH67, a green fluorescent marker, and added into the culture medium of Huh7. After coculture of M1‐derived exosomes with Huh7 for 12 h, PKH67‐labeled exosomes were internalized (Figure 5G). Furthermore, a several fold increase in exosomal H19 was observed in Huh7 (Figure 5H). And incubation with exosomes mediated hepatocyte apoptosis in vitro (Figure 5I). Our results showed that M1 secreted exosomal H19.

### 2.6 M1‐secreted exosomes exacerbated ConA‐induced hepatitis via hepatocyte apoptosis

**FIGURE 6:**
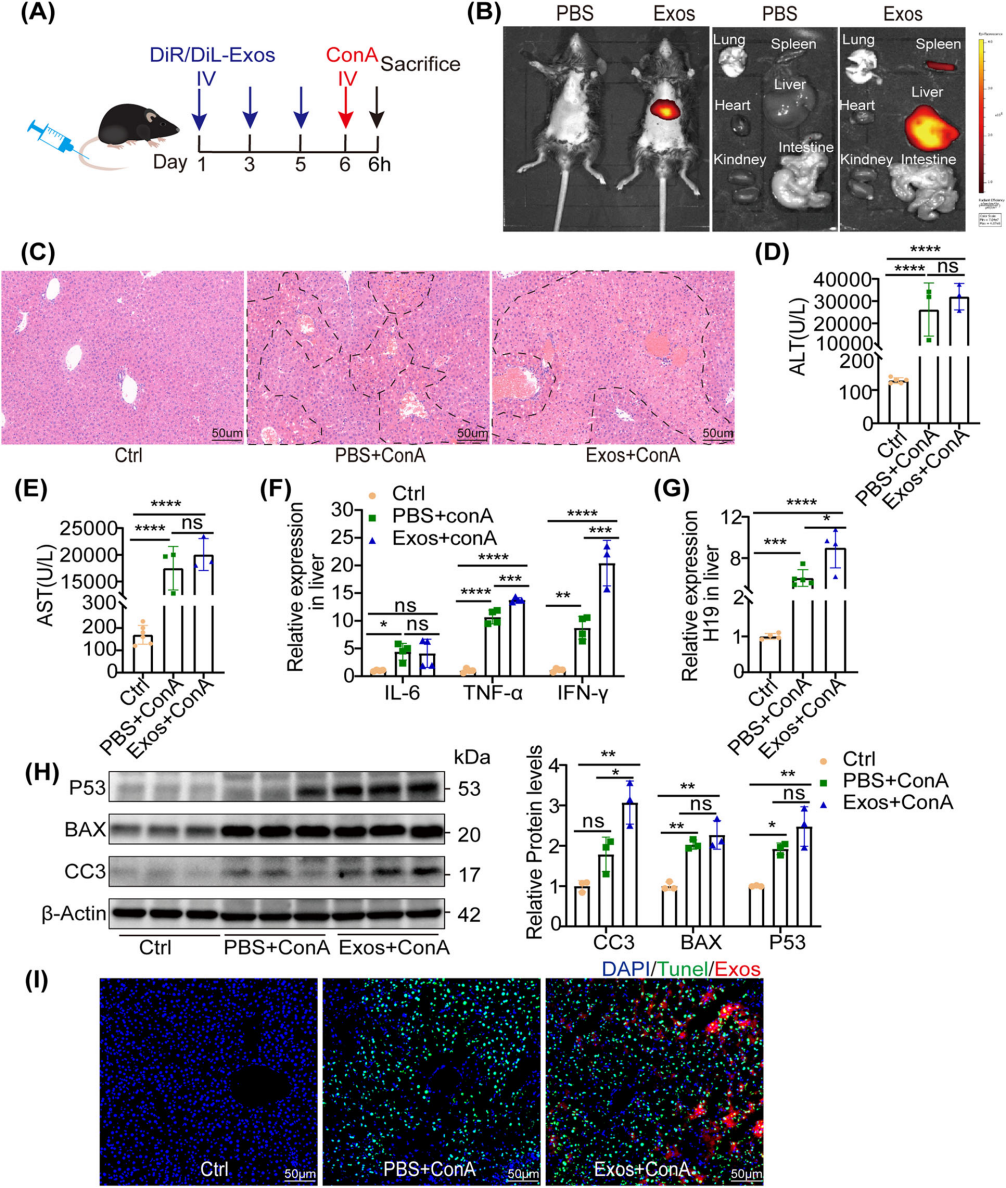
M1‐derived exosomes aggravated ConA hepatitis via increased hepatocyte apoptosis in vivo. (A) Schematic illustration of the experimental procedure analyzing the effects of M1‐derived exosomes (Exos) on ConA‐induced hepatic injury in mice receiving about 200 μg exosomes or PBS in 200ul twice a day for three times via the tail vein. (B) In vivo and ex vivo fluorescence imaging by IVIS Spectrum system was performed 4 h after DiR labeled exosomes or PBS injection. (C) Representative images showing hepatic injury (scale bar = 50 μm; the dashed line showed necrotic area). (D and E) The serum levels of ALT and AST in ConA‐treated mice injected with either PBS or exosomes (*n* = 3–5). (F) The mRNA levels of inflammatory cytokines were shown by RT‐PCR (IL‐6, TNF‐α, and IFN‐γ) (*n* = 3–5). (G) The H19 expression of liver from ConA‐treated mice was detected with RT‐PCR, after transferred exosomes (*n* = 3–5). (H) Western blot analysis of apoptotic proteins, including cleaved caspase‐3 (CC3), BAX, and p53. (I) TUNEL staining (green) in the liver was detected using immunofluorescence staining, scale bar = 50 μm (nuclei‐DAPI, exos‐DiL). Statistical analysis showed the mean ± SD; ns: not statistically significant, *p < 0.05, *p < 0.05, **p < 0.01, ***p < 0.001.

To further evaluate if exosomes derived from M1 contributed to ConA‐induced hepatitis, exosomes were isolated from RAW246.7, which was stimulated by LPS and IFN‐γ simultaneously, and transplanted via the tail vein (Figure 6A). To assess the distribution of M1‐derived exosomes, exosomes were labeled with DiR, a lipid‐based fluorescent dye. The fluorescence signals in vivo and ex vivo both accumulated in the liver (Figure 6B). Hematoxylin–eosin (H&E) staining displayed more necrotic areas and extensive hemorrhage (Figures 6C and S7A), and the serum ALT and AST levels were also slightly increased after exosome transplantation compared with ConA administration alone (Figures 6D and E). Meanwhile, exosome treatment resulted in an increase in inflammatory cytokine levels (Figure 6F), H19 expression (Figure 6G), as well as proapoptotic markers (Figure 6H). Moreover, the DiL‐labeled exosomes accumulated in TUNEL‐positive cells, as revealed by immunofluorescence microscopy (Figures 6I and S7B). Collectively, these data suggested that exosomes derived from M1 aggravated liver injury via hepatocyte apoptosis, with upregulation of H19.

### 2.7 H19 induced hepatocyte apoptosis via the HIF‐1α–p53 signaling pathway

**FIGURE 7:**
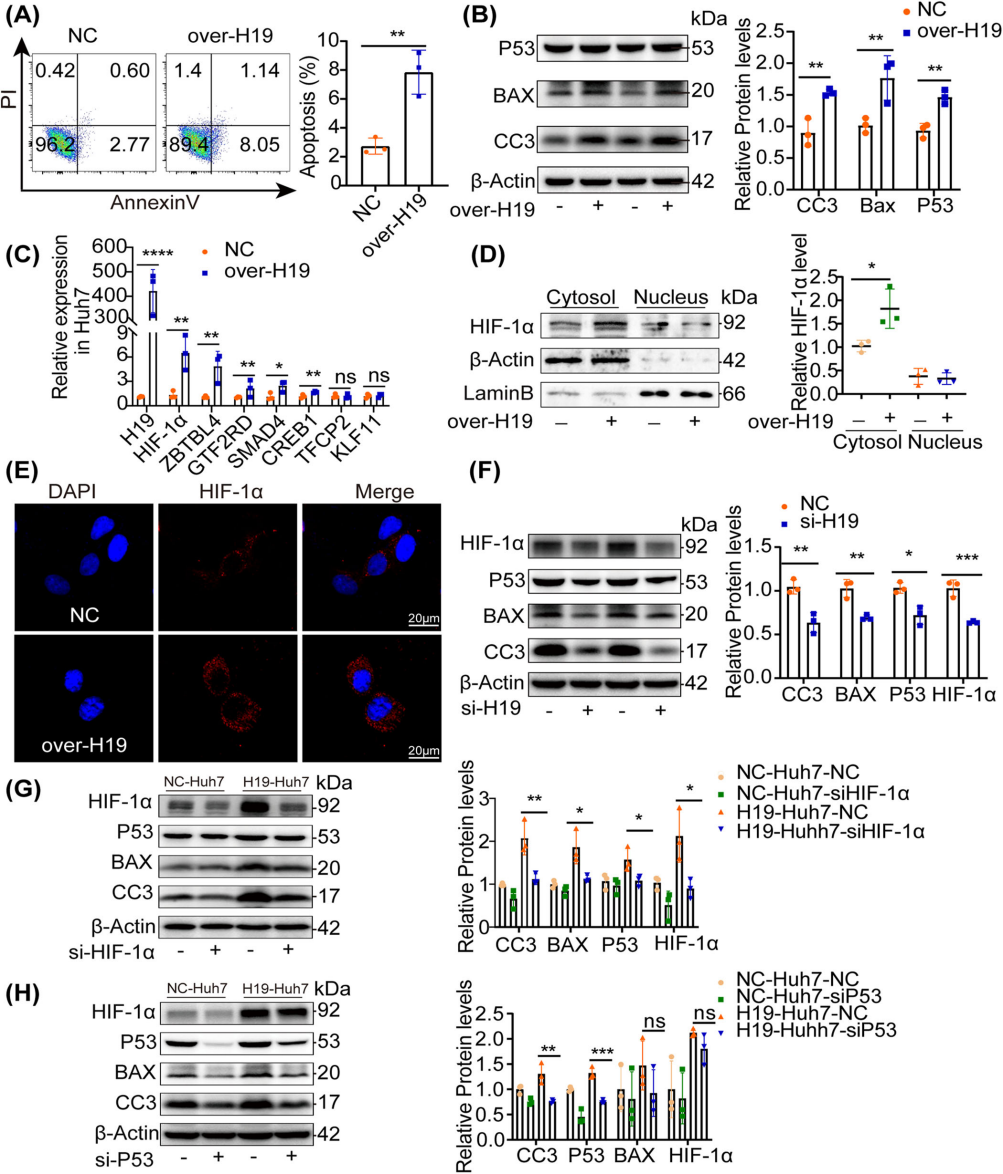
H19 regulated apoptosis in Huh7 cells via the HIF‐1α–p53 axis. (A) Flow cytometry analysis of apoptosis after transfection with H19 or scramble in Huh7 cells. (B) Immunoblots analysis of cleaved caspase‐3 (CC3), BAX, and p53 in the scramble and over‐H19 Huh7 cells. (C) RT‐PCR analysis of transcription factor expression in the scramble and over‐H19 Huh7 cells. (D) Cell fractions were isolated and measured with immunoblots detecting HIF‐1α. (E) The subcellular location of HIF‐1α in the scramble or overexpressed‐H19 Huh7 cell was shown by Immunofluorescent confocal microscopy, scale bar = 20 μm. (F) Immunoblots analysis of HIF‐1α, p53, and CC‐3 in Huh7 cells transfected with siH19. (G‐H) Immunoblots analysis of p53, and CC‐3 in NC/H19 Huh7 cells transfected with siHIF‐1α or sip53. Statistical analysis showed the mean ± SD; ns: not statistically significant, *p < 0.05, *p < 0.05, **p < 0.01, ***p < 0.001.

To further explore the mechanisms by which H19 induced hepatocyte apoptosis, we observed overexpressed H19 significantly increased apoptosis in Huh7 (Figures 7A and S8A), and proapoptotic markers levels, including p53, CC3, and BAX (Figure 7B). Transcription factors were considered master regulators, including gene expression, chromatin stability, and cell homeostasis.^19^ The online bioinformatics tool (PROMO) was applied to predict the targets of H19 (Figure S8B). And, several potential transcriptional factors mediated by H19 were reported to regulate apoptosis.^20^ Among them, HIF‐1α was the most differentially regulated factor (Figure 7C). HIF‐1α nuclear translocation is a key step for activating the target gene, which regulated metabolism, angiogenesis, and cell survival.^21^ We examined the subcellular localization of HIF‐1α in Huh7 by the immunofluorescence and western blot of cell fractions, and found HIF‐1α accumulation in the cytoplasm after H19 overexpression (Figures 7D and E). To determine the relationship between HIF‐1α and H19, H19‐silenced in Huh7 showed a significant decrease in apoptosis and the level of HIF‐1α (Figures 7F and S8C). HIF‐1α was found to regulate apoptosis through p53 upregulation.^22^ After HIF‐1α was knocked down, we found the expression of p53 decreased in over‐H19 Huh7, as well as apoptosis, without feedback on H19 (Figures 7G and S8D and E). In addition, silenced p53 in over‐H19 Huh7 decreased apoptosis, while HIF‐1α expression remained unchanged (Figure 7H). Liver‐specific overexpression or knockdown of H19 in mice upregulated and reduced HIF‐1α expression (Figures S10G and S11). Collectively, these data revealed that H19 regulated apoptosis in Huh7 cells via the HIF‐1α–p53 axis.

## 3 DISCUSSION

ConA‐induced hepatitis is characterized by T cell activation‐mediated AIH involving Kuffer cells/macrophages, NKT, and NK cells.^23^ H19 is an imprinted and maternally expressed lncRNA, which is highly expressed in the fetal liver but repressed after birth and in the adult liver.^24^ Recent studies have demonstrated H19 increased in the liver of patients with fibrosis and cirrhosis, regardless of the underlying disease.^25^ Our studies also observed that H19 was noticeably increased in female mice, not in male mice after ConA administration (Figure S12), and aberrant expression of H19 aggravated liver injury via hepatic apoptosis.

Nevertheless, the underlying mechanisms of H19 in liver injury remain unclear. H19 was markedly upregulated in primary sclerosing cholangitis and primary biliary cirrhosis, which prevented zinc finger E‐box binding homeobox 1 (ZEB1) inhibition of epithelial cell adhesion molecule (EpCAM), causing EpCAM activation and contributing to biliary hyperplasia.^26^ In addition, H19 was highly expressed in cholangiocyte‐derived exosomes, which were transferred into hepatocytes and led to dysregulation of hepatic bile acid metabolism.^17^ Intriguingly, our studies showed that treatment with GW4869 significantly ameliorated ConA‐induced hepatitis and reduced H19 levels both in the liver and hepatocytes.

Previous studies indicated that Kupffer cells also played a critical role in ConA‐induced hepatitis.^27^, ^28^ After administration of ConA, activated Kupffer cells produced and released reactive oxygen species, which were the main effectors of hepatotoxicity.^29^ Activated Kupffer cells also secreted TNF‐α, IL‐12, and IL‐18. Moreover, T cells were activated, which produced IFN‐γ and activated Kupffer cells in a positive feedback loop..^30^ The proportion of activated Kupffer cells significantly increased at 1 h and gradually decreased between 3 and 6 h following ConA administration,^5^ which was consistent with our results, accompanied by H19 upregulation.

Moreover, the specific depletion of the macrophages by GdCl3 or clodronate liposomes completely reversed liver injury.^31,^ ^32^ Macrophage depletion by clodronate liposomes inhibited ConA‐induced hepatic apoptosis, accompanied by inhibition of H19 upregulation. These results suggest that macrophage‐derived H19 plays a critical role in promoting ConA‐induced liver injury. Recent studies also demonstrated H19 was colocalized with F4/80^+^ in the cytoplasm.^14^ Our results showed M1‐derived H19 induced hepatocyte apoptosis via exosomes in vivo and in vitro. Nevertheless, further studies are required to investigate the mechanisms underlying H19 upregulation in macrophages and whether the exosomal H19 was transported into immune cells.

H19 recruited transcription factors, such as Foxo1^33^ and E2F,^34^ and promoted phosphorylation or nuclear translocation of transcription factors^35^ to regulate the target genes. We found that H19‐overexpressed in the liver upregulated the level of HIF‐1α (Figure S11). And H19‐knocked down in the liver alleviated ConA‐induced liver injury, and downregulated the levels of HIF‐1α, p53, and CC3 (Figure S10). Furthermore, in vitro, H19 interacted with HIF‐1α and detained it in the cytoplasm. The increased cytoplasmic HIF‐1α prevented the reduction of p53, which contributed to elevated BAX and CC3 in the abdominal aortic aneurysm model.^20^ Matouk et al. reported hypoxia significantly upregulated H19 in tumor cells, and HIF‐1α was regarded as a critical role during this induction.^36^, ^37^ In our experiments, blocking HIF‐1α in Huh7 cells did not induce change in H19 expression. Another study reported that H19 upregulation only occurred in p53 mutated cells and most severely in p53 null cells under hypoxia.^38^

In this work, we identified that H19 was expressed in M1‐derived exosomes, however, we cannot identify that the M1‐derived exosomal H19 was transported into hepatocytes in the liver post‐ConA injection. Although administration of ConA has been widely used to model immune‐mediated liver injury in mice, mimicking clinical features of human AIH, the mechanisms remain to be determined in AIH.

In conclusion, we identified the role of H19 in ConA‐induced hepatitis, which was derived from M1 and transported into hepatocytes via exosomes, contributing to hepatocyte apoptosis via the HIF1α‐p53 signaling pathway. The findings represent a novel mechanism of ConA‐induced hepatitis.

## 4 MATERIALS AND METHODS

### 4.1 Tissue specimens

The liver tissues were collected from AIH and hepatocellular carcinoma patients who were first diagnosed and underwent surgical resection at the First Affiliated Hospital of Zhejiang University. The studies involving human participants were reviewed and approved by the Clinical Research Ethics Committee of The First Affiliated Hospital, School of Medicine, Zhejiang University (Approval notice 2021−29). The patients/participants provided their written informed consent to participate in this study. Written informed consent was obtained from the individual(s) for the publication of any potentially identifiable images or data included in this article.

### 4.2 Animal treatment

Six to eight weeks‐old female C57BL/6 mice were purchased from the Laboratory Animal Center of Shanghai SLRC Experimental Animal Company Ltd. (Shanghai, China). The experiments were approved by the Animal Care and Use Committee of the medical college of Zhejiang University (Ethics number: 2021‐06). To induce acute hepatitis, mice were injected with a single dose of ConA (Sigma–Aldrich, St. Louis, MO) at 10 μg/g through the tail vein. Mice were intravenously injected with 5 × 1011 pfu AAV particles (Vigene, Jinan, China). In addition, GW4869 (2.5 μg/g, S7609; SELLECK) was intraperitoneally injected every other day for two weeks.^39^, ^40^ To deplete liver macrophages, 200 μL of Clodronate Liposomes (Yeasen; 40337ES08) was intraperitoneally injected 24 h before ConA injection.

### 4.3 Isolation of hepatic MNCs and flow cytometry analysis

The isolation of hepatic MNCs was performed as previously described.^41^ Isolated MNCs were incubated with antibodies against F4/80 (PE‐Cy7; Biolegend), CD206 (APC; Biolegend), CD11c (FITC; Biolegend), CD45 (PERCP; Biolegend), TCR β (APC‐Cy7; Biolegend), and NK1.1 (APC; Biolegend). The population of macrophages and T cells was purified using a BD FACS Aria II flow cytometer (BD Biosciences).

### 4.4 Cell culture and reagents

The human liver cancer cell lines Huh7 (ATCC) was maintained in Dulbecco's modified Eagle medium (Sigma, USA), and the human monocytic cell line THP‐1 cells (ATCC) and the murine macrophage cell line Raw 264.7 (ATCC) were cultured in PRIM‐1640 medium (Sigma). THP‐1 cells were plated in PRIM‐1640 and phorbol 12‐myristate 13‐acetate (PMA) (10 ng/mL) (HY‐18739; MCE, USA) for 24 h. Raw 264.7 macrophages or PMA‐induced THP‐1 cells were then stimulated with 100 ng/mL lipopolysaccharide (LPS) (S1735; Beyotime) and 20 ng/mL IFN‐γ (570206; PeproTech, USA).^42^, ^43^

### 4.5 Exosome isolation, qualification, and characterization

THP‐1 cells were stimulated with LPS /IFN‐γ (PeproTech, 300−02) without fetal bovine serum for 48 h; the supernatant was collected, and exosomes were isolated as previously described.^44^ The exosomes were characterized by exosome‐specific markers TSG101 (#28283‐1‐AP; Wuhan), Alix (#12422‐1‐AP; Wuhan), and CD63 (#25682‐1‐APl Wuhan) by western blot. Furthermore, a BCA Protein assay kit (PC0020; Solarbio) was used for exosome quantification. To monitor exosome uptake, the exosomes were labeled with PKH67 (MIDI67; Sigma‐Aldrich).

### 4.6 Exosome tracking in vivo

To visualize the distribution of exosomes in vivo, the purified exosomes (about 1 μg/μL at protein level) were incubated with 1 mM DiL (Thermo #D282) or DiR (Invitrogen; D12731) at a volume ratio of 500:1 for 30 min in the dark, then washed in PBS, and centrifuged again. Next, mice at 6 weeks were injected with DiR‐labeled exosomes via the tail vein (dosage: 200 μg exosome in 200 μL of PBS/ per mouse). Four hours after injection, the distribution of exosomes in mice and tissues was assessed by the In Vivo Imaging Systems (IVIS) Spectrum system (PerkinElmer, USA).

### 4.7 Co‐culture assay

Subsequently, PMA‐induced THP‐1 cells were stimulated with LPS and IFN‐γ for 24 h, and then cultured with Huh7 cells. The Huh7 cells (1 × 10^5^/well) were placed in the lower chamber, while THP‐1 cells (5 × 10^5^/well) were placed in the upper chamber, separated by a trans‐well plate (0.4 μm polycarbonate filter; Corning). After 24 h, Huh7 cells were collected for further experiments.

### 4.8 Histopathological staining and analysis

H&E stained sections (4 μm) were evaluated independently by two pathologists blind to the study groups according to the Ishak modified HAI score.^45^ Liver specimens were incubated with primary antibodies, including F4/80 (1:1000; Servicebio), CD63 (1:500; Servicebio), and CC3 (1:500; Servicebio), and then incubated with secondary antibodies, including anti‐mouse or rabbit immunoglobulin G. Subsequently, a TUNEL assay kit (Servicebio; G1501) was used to assess apoptosis. The Tunel, CD63, and F4/80 stained areas were calculated via Image‐Pro Plus.

### 4.9 Fluorescence in situ hybridization

Fluorescence in situ hybridization (FISH) was performed as previously described.^46^ The hybridization signals were detected with the Fluorescent in Situ Hybridization Kit (GenePharma, Shanghai, China).

### 4.10 Immunoblotting

Western blotting was performed as previously described.^47^ Primary antibodies p53 (CST; #2524), BAX (CST; #41162), HIF‐1α (Abcam; ab179483), CC3 (Abcam; ab2302), and CD63 (Abcam; ab52090) were diluted to a 1:1000 ratio.

### 4.11 Real‐time PCR analysis

The isolated RNA was reverse‐transcribed to cDNA with TransScript II Q (Vazyme). Real‐time PCR (RT‐PCR) was performed with SYBR qPCR Master Mix (Vazyme) and QuantStudio 5 (Applied Biosystems) was used for amplification. The primers were designed by the online website of NCBI. The 5′–3′ sequences of primer pairs were as follows: mouse‐H19: CCTTGTCGTAGAAGCCGTCTGTTC(F) and AGGATGATGTGGGTGGTGGTCTC (R); mouse‐IL‐6: TGATGGATGCTACCAAACTGGA (F) and TGTGACTCCAGCTTATCTCTTGG (R); mouse‐TNF‐α: CCCTCACACTCACAAACCAC (F) and ACAAGGTACAACCCATCGGC (R); mouse‐IFN‐γ: AGACAATCAGGCCATCAGCAA(F) and TGTGGGTTGTTGACCTCAAACT (R); mouse‐GAPDH: CATCACTGCCACCCAGAAGACTG (F) and ATGCCAGTGAGCTTCCCGTTCAG (R); mouse‐Actin :CATTGCTGACAGGATGCAGAAGG (F) and TGCTGGAAGGTGGACAGTGAGG (R); human‐H19: ACGTGACAAGCAGGACATGACA(F) and ACCAGCCTAAGGTGTTCAGGAA(R); human‐HIF‐1α: AGGTTGAGGGACGGAGATTT (F) and TGGCTGCATCTCGAGACTTT (R); human‐Bax: TCATGGGCTGGACATTGGAC (F) and GCGTCCCAAAGTAGGAGAGG (R); human‐Bcl2: GAACTGGGGGAGGATTGTGG (F) and ACTTCACTTGTGGCCCAGAT(R); human‐Actin GTGGCCGAGGACTTTGATTG(F) and CCTGTAACAACGCATCTCATATT(R); human‐GAPDH: CTCTGCTCCTCCTGTTCGAC(F) and ACGACCAAATCCGTTGACTC(R); human‐KLF11: ATGCACACGCCGGACTT(F) and CAGGCGTGAGGGGTCTTATC(R); human‐SP1 CCACCATGAGCGACCAAGAT(F) and AAGGCACCACCACCATTACC(R); human‐CREB1: GTGTGTTACGTGGGGGAGAG(F) and GCATCTCCACTCTGCTGGTT(R).

### 4.12 Transfection

The plasmids for H19, siH19, siHIF‐1α, and sip53 for RNA overexpression or knockdown were designed and synthesized by GenePharma (Shanghai, China). The transfections were carried out with Lipofectamine 2000 (Invitrogen). The lentivirus vectors (Lv‐GV531) were designed by Genechem (Shanghai, China), which were then selected with puromycin to yield a stable H19‐overexpressed Huh7 cell line.

### 4.13 Isolation of nuclear and cytoplasmic protein

After Huh7 cells were transfected with H19, cytoplasmic and nuclear fractions were isolated using the PARIS kit (AM1921; Thermo Fisher Scientific). Cell fractions were then analyzed by Western blotting. β‐actin (Gene Script; A00702) and Lamin B1 (ab133741; Abcam) were used as the cytoplasmic and the nuclear loading control, respectively.

### 4.14 Annexin V‐PI apoptosis assay

Cells were collected with a specific treatment and were then evaluated by an Apoptosis Assay Kit (APCC101; Multi Sciences). The cells were analyzed by flow cytometry with BD FACS Calibur (BD Biosciences, San Jose, CA, USA).

### 4.15 Statistical analysis

All data were performed with GraphPad Prism 8.0 software (GraphPad Software, San Diego, CA). Statistical significance between the two groups were analyzed by using Student's *t*‐test. Comparisons between more than two groups were analyzed by using one‐way or two‐way ANOVA. The Kaplan–Meier curve was used for evaluating the overall survival. All data are presented as mean ± standard deviation (SD), and at least three independent experiments. *p* < 0.05 was considered statistically significant. *p ≤ 0.05, **p < 0.01, ***p < 0.001, ****p≤ 0.0001, ns: *p* > 0.05.

## AUTHOR CONTRIBUTION

Yongting Zhang and Hongyan Diao conceived and designed the study. Yongting Zhang, Liang Hong, Xuehui Li, Yuyu Li, Xujun Zhang, and Jingjing Jiang performed the experiments and statistical analysis. Yongting Zhang, Jingjing Jiang, and Fan Shi created the figures. Yongting Zhang and Hongyan Diao wrote and edited the manuscript. All authors have read and approved the final manuscript.

## CONFLICT OF INTEREST STATEMENT

The authors declare that they have no competing interests.

## ETHICS STATEMENT

The studies involving human participants were reviewed and approved by the Clinical Research Ethics Committee of The First Affiliated Hospital, School of Medicine, Zhejiang University (Approval notice 2021−29). The patients/participants provided their written informed consent to participate in this study. Written informed consent was obtained from the individual(s) for the publication of any potentially identifiable images or data included in this article.

## Supporting information

Supporting Information

## Data Availability

Additional data collected during this study are available from the corresponding authors upon reasonable request.
