# Supplementary material for: M1 macrophage‐derived exosomes promote autoimmune liver injury by transferring long noncoding RNA H19 to hepatocytes
Source: MedComm (2020). 2023 Jun 29;4(4):e303. doi: 10.1002/mco2.303 (PMC10310975; doi:10.1002/mco2.303)
Supplement: Supplementary file 1 — Supporting Information [file MCO2-4-e303-s001.docx]

**M1 Macrophage-derived exosomes promote autoimmune liver injury by transferring long noncoding RNA H19 to hepatocytes**

Yongting Zhang ^1^, Liang Hong ^1^, Xuehui Li ^1^, Yuyu Li ^1^, Xujun Zhang ^1^, Jingjing Jiang ^1^, Fan Shi ^1^, Hongyan Diao ^1,^ *

^1^State Key Laboratory for Diagnosis & Treatment of Infectious Diseases, National Clinical Research Center for Infectious Disease, Collaborative Innovation Center for Diagnosis & Treatment of Infectious Diseases, The First Affiliated Hospital, College of Medicine, Zhejiang University, Hangzhou, China

***Correspondence**

Hongyan Diao, State Key Laboratory for Diagnosis & Treatment of Infectious Diseases, National Clinical Research Center for Infectious Disease, Collaborative Innovation Center for Diagnosis & Treatment of Infectious Diseases, The First Affiliated Hospital, College of Medicine, Zhejiang University, Hangzhou, 310003, China **E-mail**：diaohy@zju.edu.cn

**Funding information**

This work was supported by the National Key Research and Development Program of China (2021YFA1301100, 2021YFA1301101), the Key Research & Development Plan of Zhejiang Province (2019C04005), the Research Project of Jinan Microecological Biomedicine Shandong Laboratory (JNL-2022012B), and the Fundamental Research Funds for the Central Universities (2022ZFJH003).


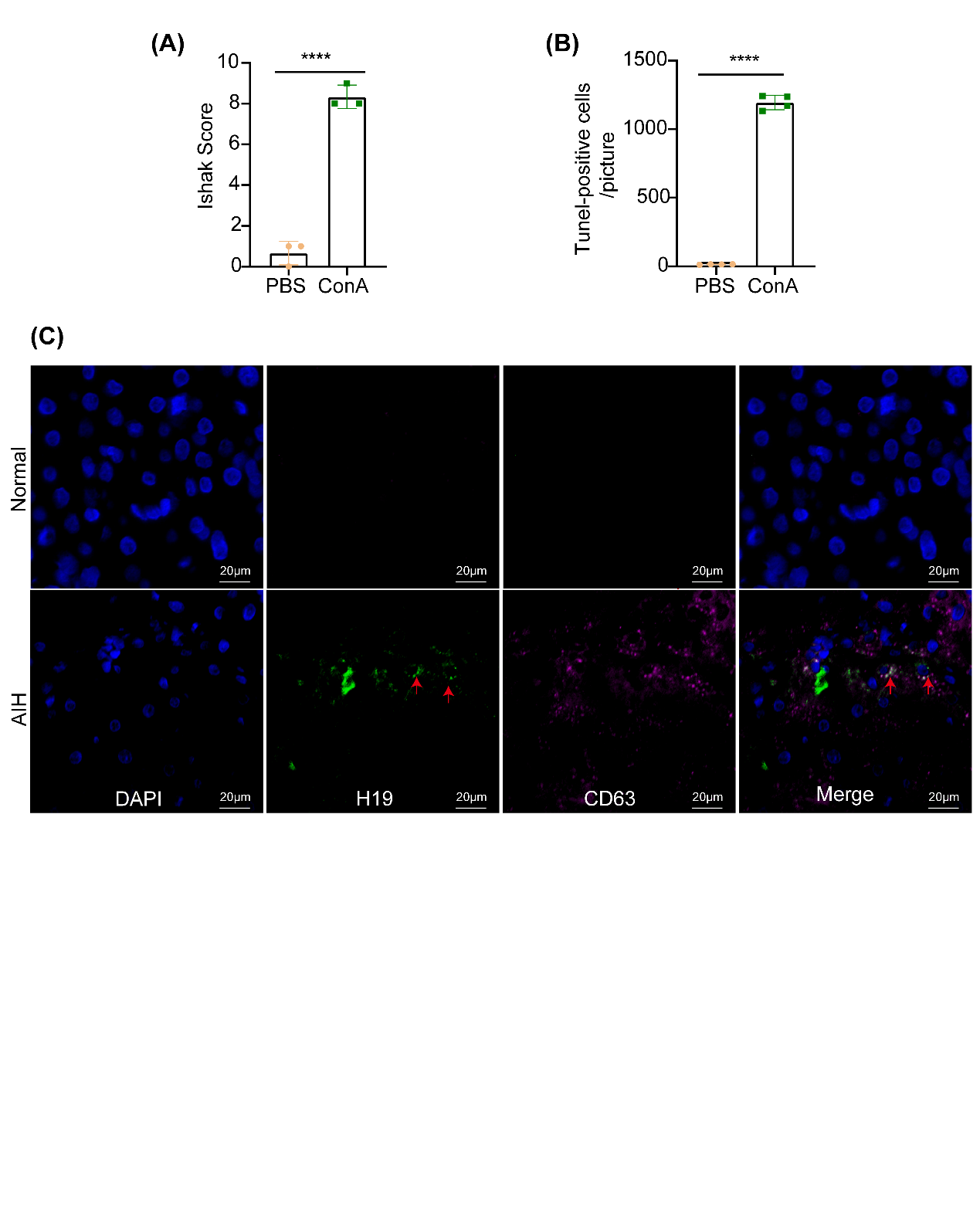


Figure S1. (A) After ConA treatment, the histology change was assessed by ISHAK scores. (B) Statistical analysis of TUNEL-positive cells (n=3). (C) Co-localization of CD63 (pink) and H19 (green) in the liver of AIH patient and Normal (arrows showed H19) (n=3), scale bar=20 μm.


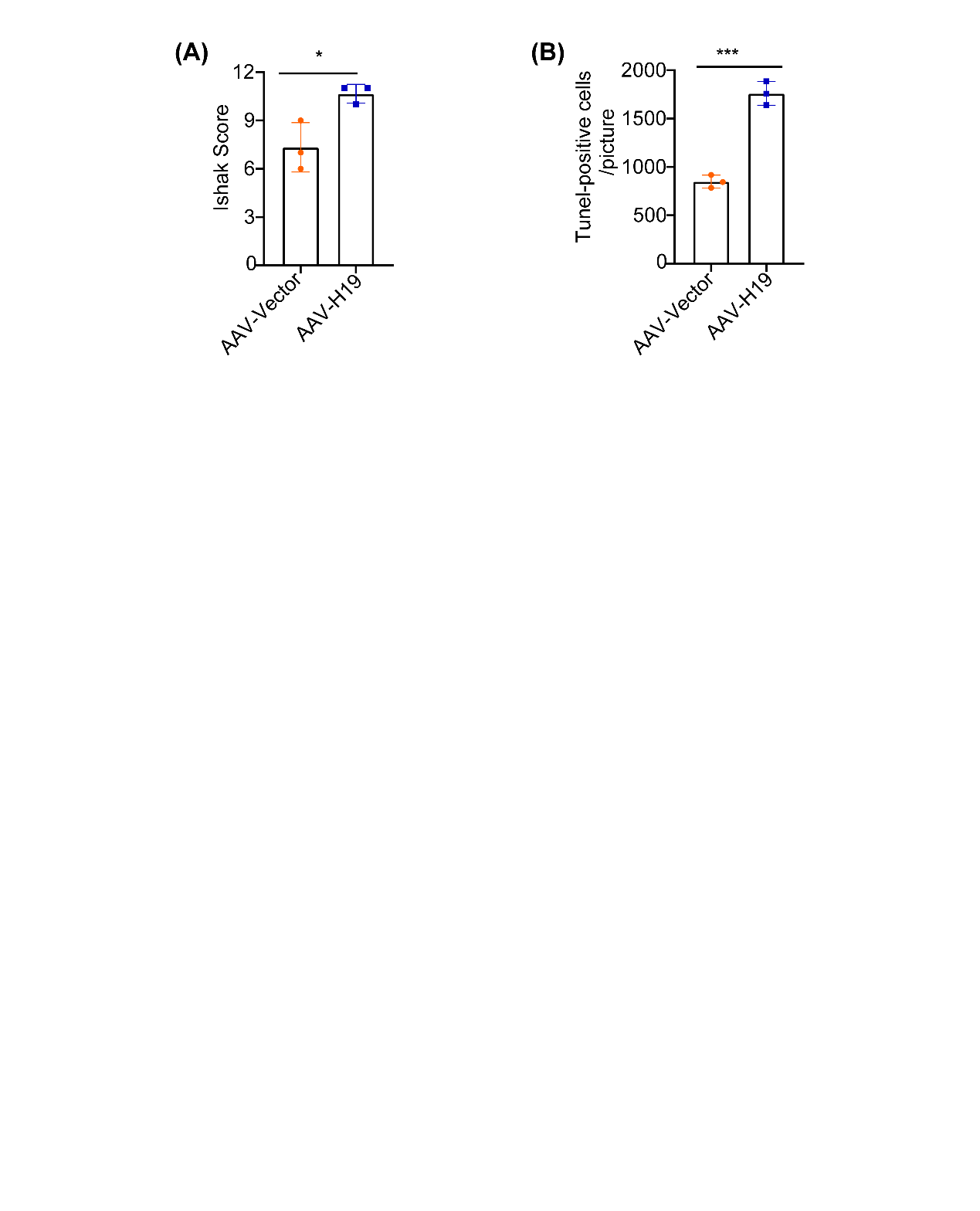


Figure S2. Overexpressed H19 aggravated ConA-induced hepatitis. (A) The histology change was assessed by ISHAK scores (n=3). (B) Statistical analysis of TUNEL-positive cells (n=3).


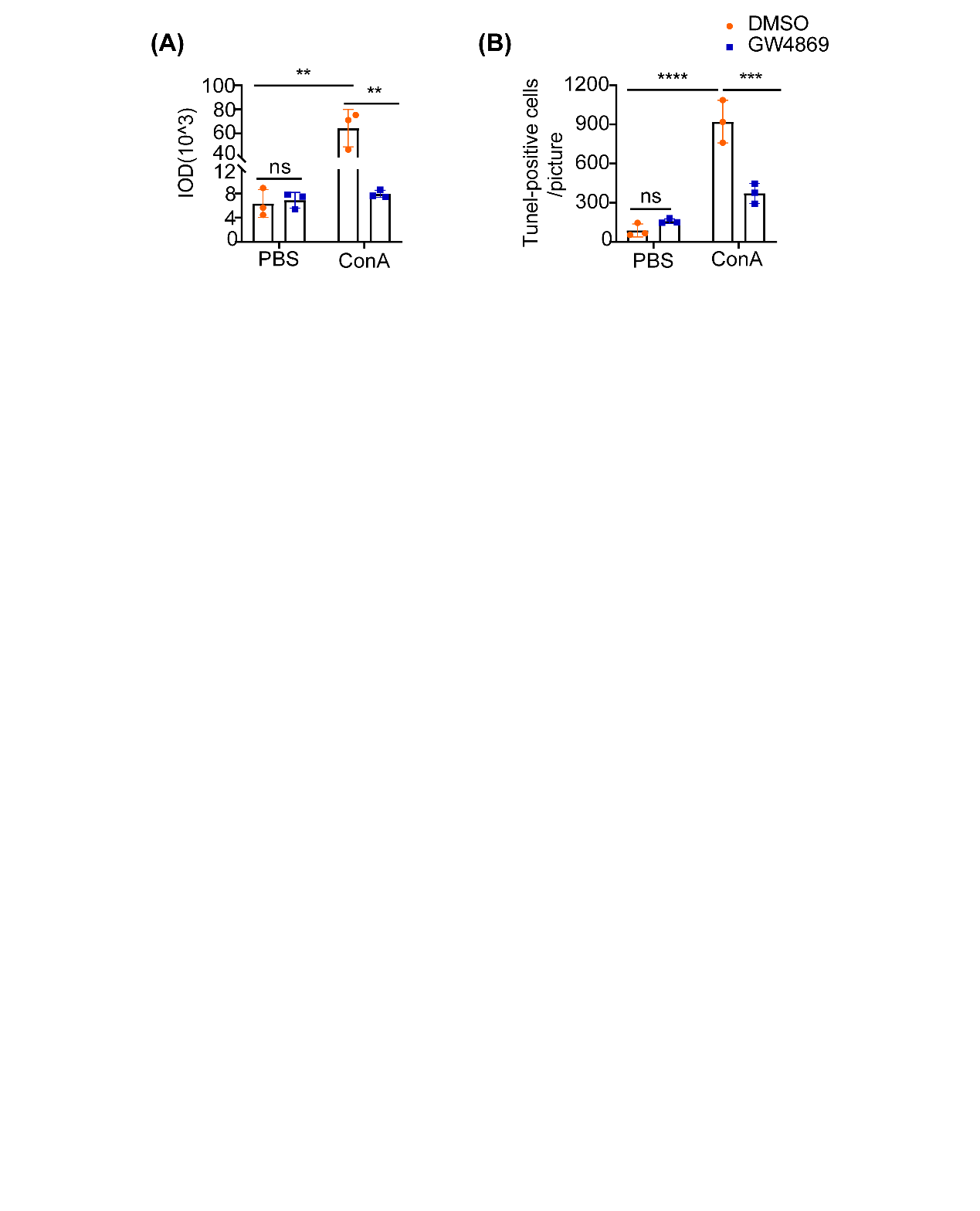


Figure S3. The exosome inhibitor GW4869 attenuated ConA-induced hepatitis. (A) The histology change was assessed by ISHAK scores (n=3). (B) Statistical analysis of TUNEL-positive cells (n=3).


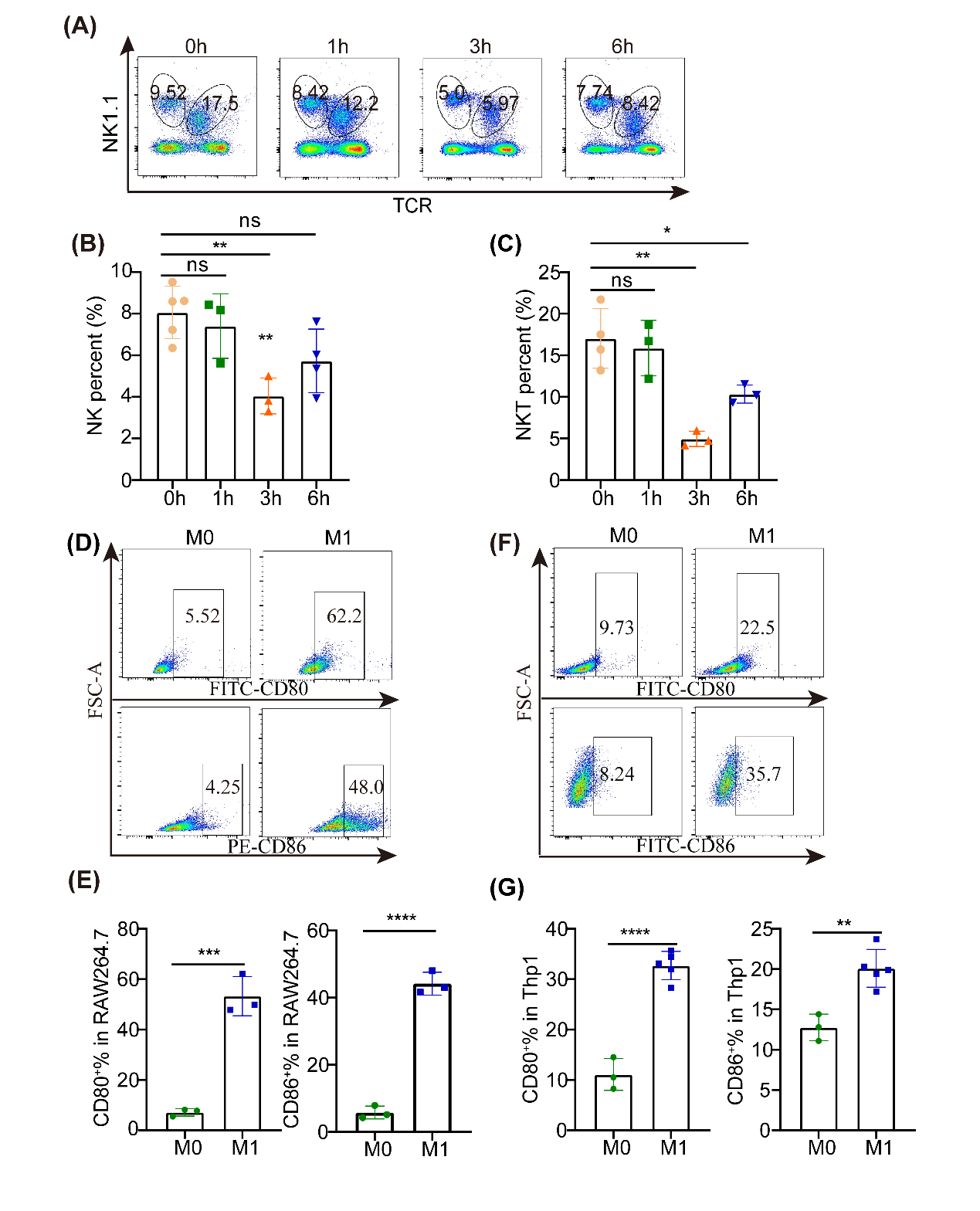


Figure S4. Flow cytometry analysis of MNCs and identification of type I macrophages. (A-C) Flow cytometry analysis of NKT and NK cells in mononuclear cells (MNCs) isolated from ConA-treated mice (n=3~5); (D-G) Flow cytometry showed the percentages of CD80 and CD86 after RAW264.7 and THP-1 were simultaneously stimulated with 20 ng/ml IFN-γ and100 ng/ml LPS for 24 h. Statistical analysis showed the mean±SD; ns: not statistically significant, *p<0.05, **p<0.01, ***p<0.001.


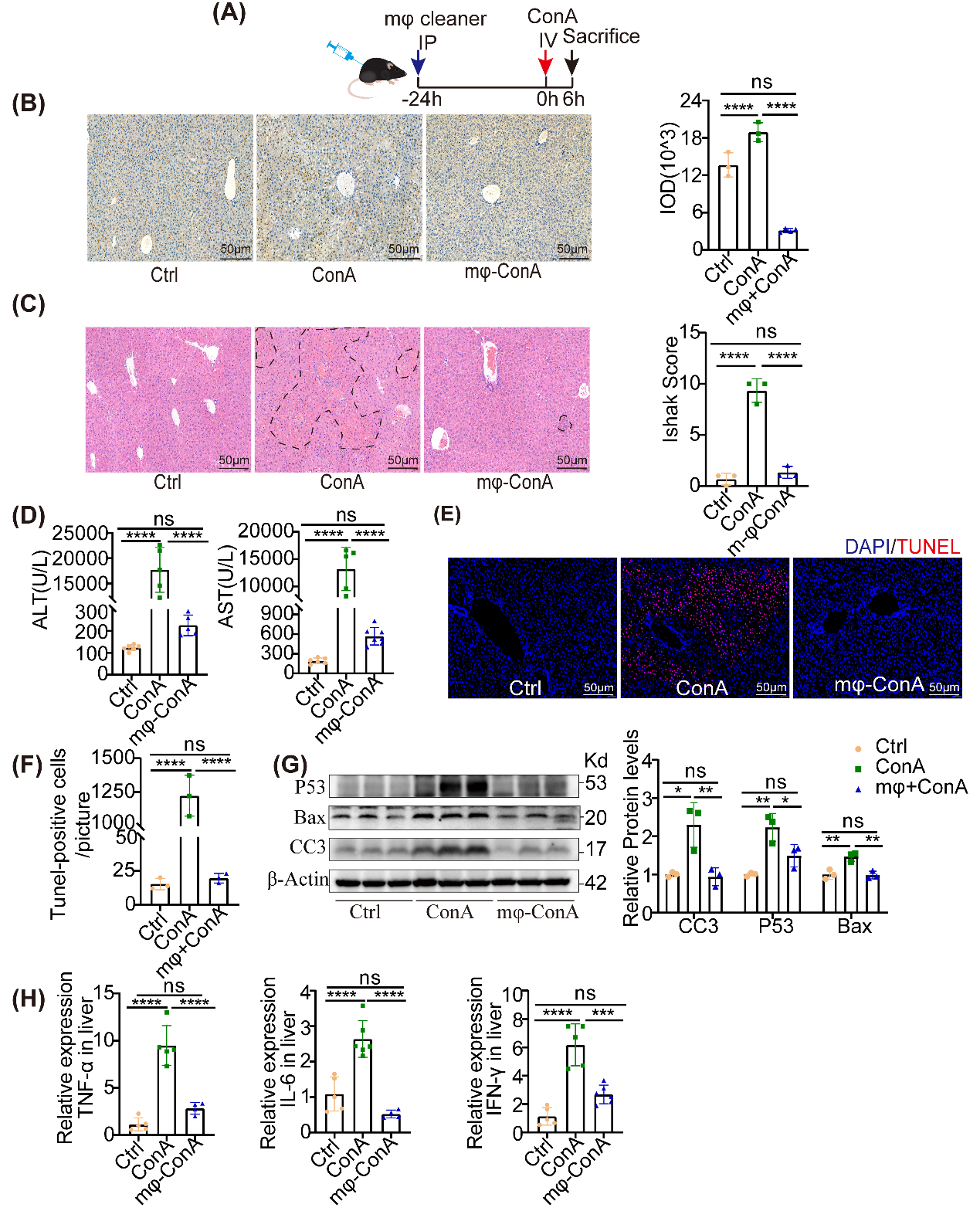


Figure S5. Depletion of macrophages alleviated ConA-induced hepatitis. (A) Clodronate was injected intraperitoneally 24h before ConA treatment. (B) Representative immunohistochemistry staining of F4/80, and statistical analysis of F4/80-positive cells. (C) Representative images of histologic liver injury, and ISHAK scores (n=3), scale bar= 50 μm (dashed line showed necrotic area). (D) The serum levels of ALT and AST after injection with ConA with or without clodronate liposome (n=5~6). (E) Representative images of TUNEL staining, scale bar=50 μm. (F) Statistical analysis of TUNEL-positive cells (n=3). (G) Western blot analysis of pro-apoptotic markers. (H) RT-PCR detected the mRNA levels of inflammatory markers, including IL-6, TNF-α, and IFN-γ. Statistical analysis showed the mean±SD; ns: not statistically significant, *p<0.05, **p<0.01, ***p<0.001.


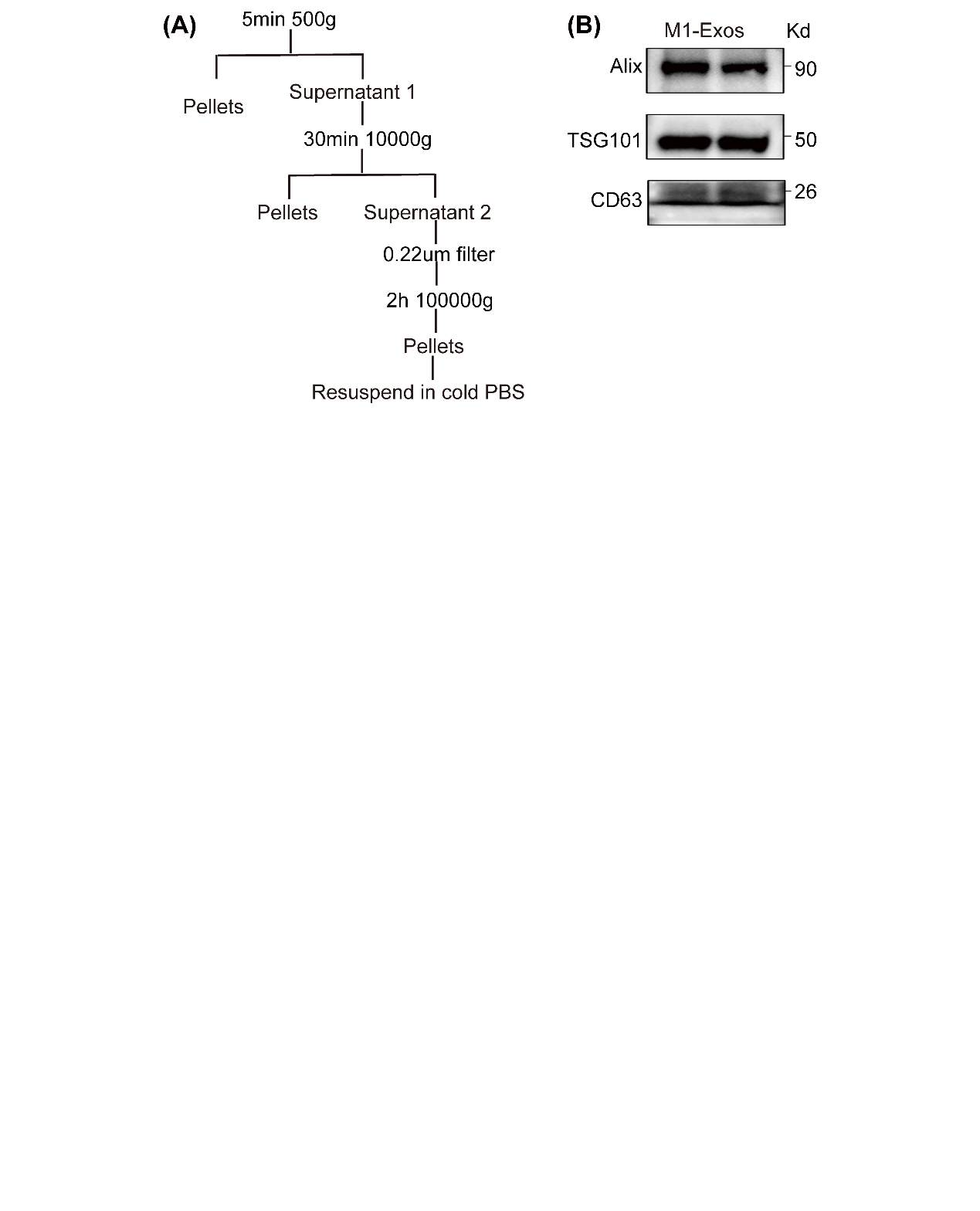


Figure S6. Identification of exosomes derived from M1 cells. (A) Schematic illustration of the exosome acquisition by standard differential ultracentrifugation. (B) Western blot analysis of exosomal proteins (CD63, TSG101, Alix).


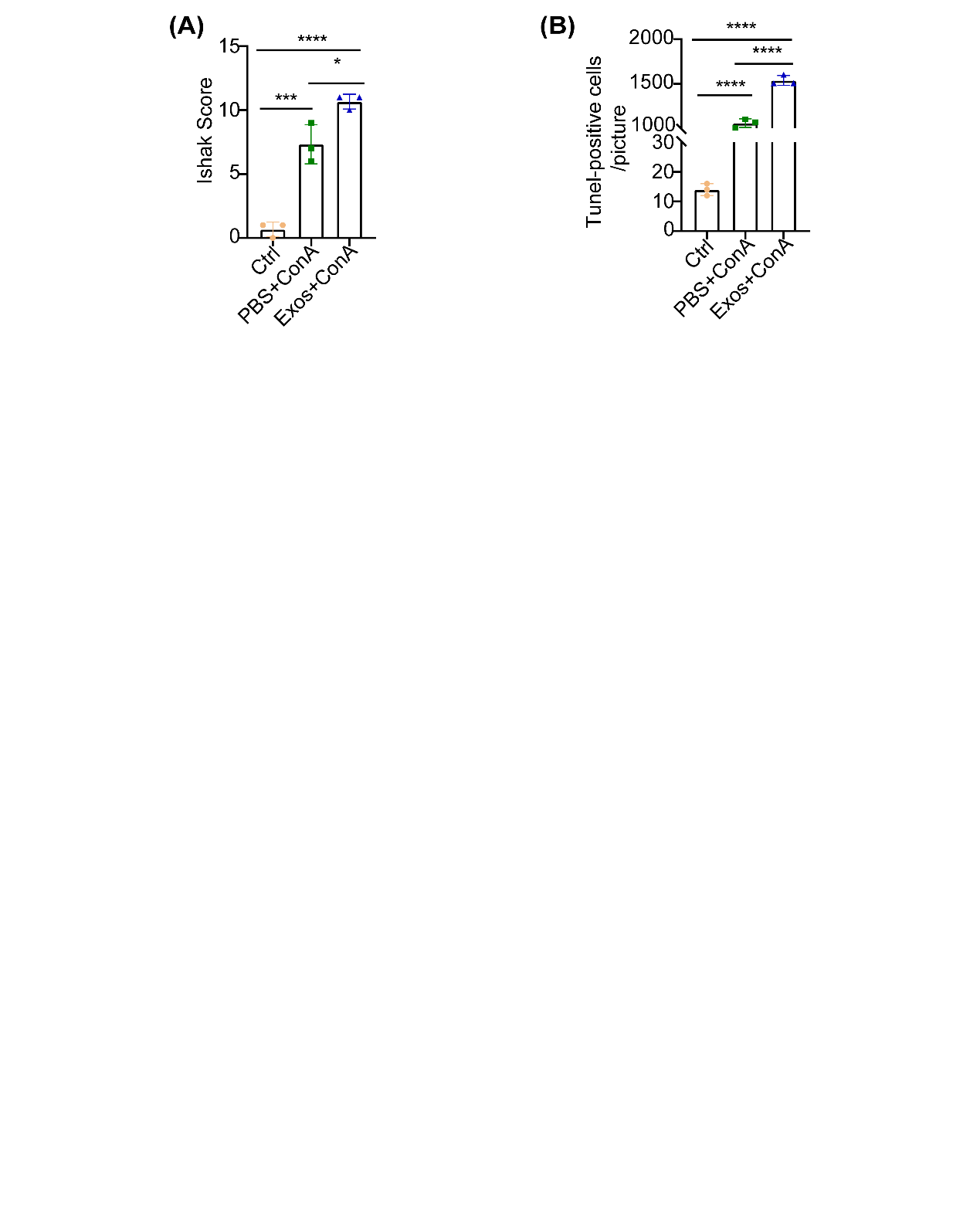


Figure S7. M1-derived exosomes aggravated ConA-induced hepatitis via increased hepatocyte apoptosis in vivo. (A) The histology change was assessed by ISHAK scores. (B) Statistical analysis of TUNEL-positive cells (n=3).


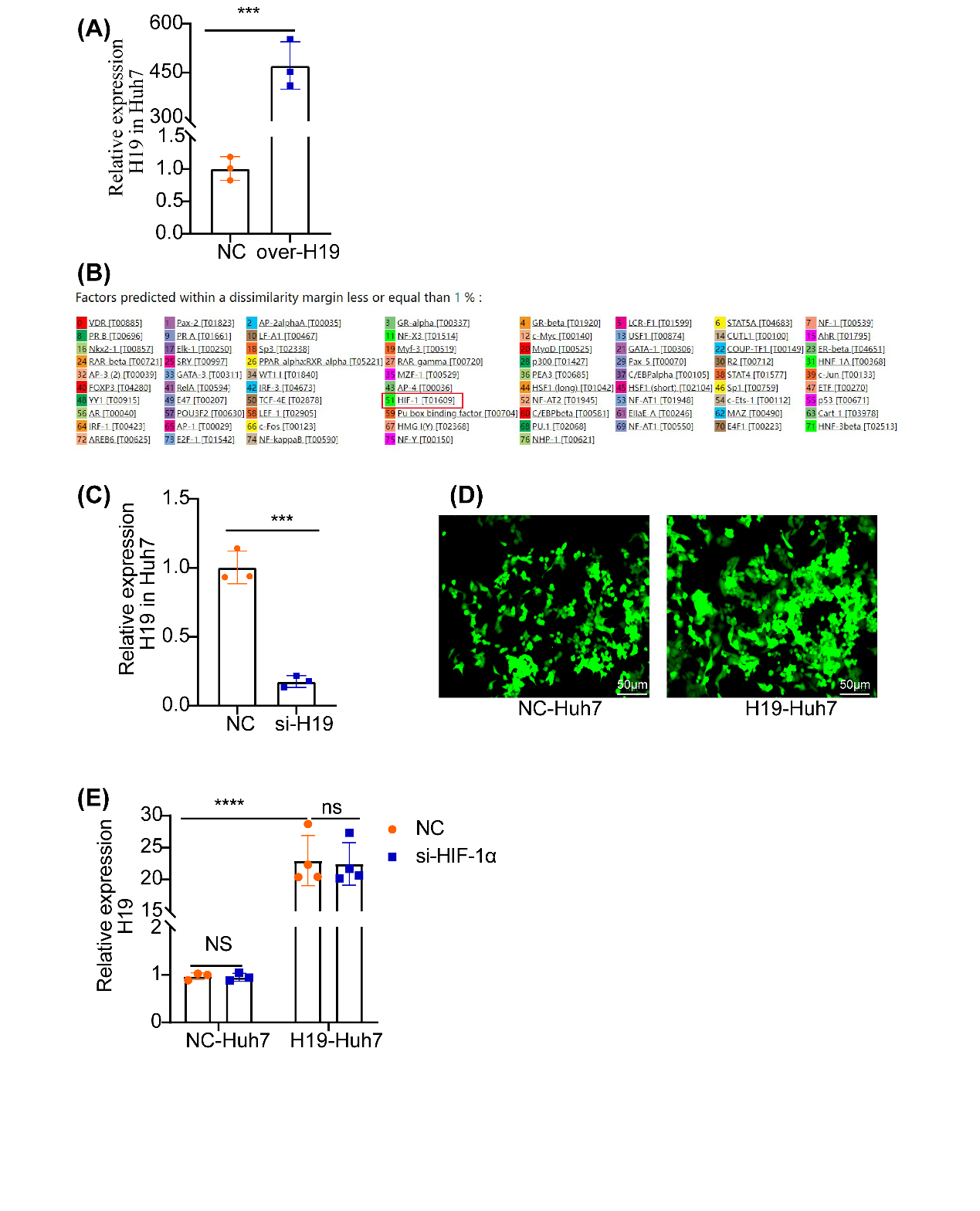


Figure S8. H19 regulated apoptosis in Huh7 cells via the HIF1а-P53 axis. (A) RT-PCR analysis of H19 expression in Huh7 cells after H19 overexpression. (B) Transcription factors of H19 were predicted by ALGGEN. (C) RT-PCR analysis of H19 expression in Huh7 cells following siH19 treatment. (D) GFP was expressed in the stable cell line over-H19-Huh7, which was successfully constructed after selection with puromycin for 2~3 weeks, scale bar=50 μm. (E) RT-PCR analysis of H19 expression in overHuh7 cells after siHIF1а treatment. Statistical analysis showed the mean±SD; ns: not statistically significant, *P<0.05, **P<0.01, ***P<0.001.


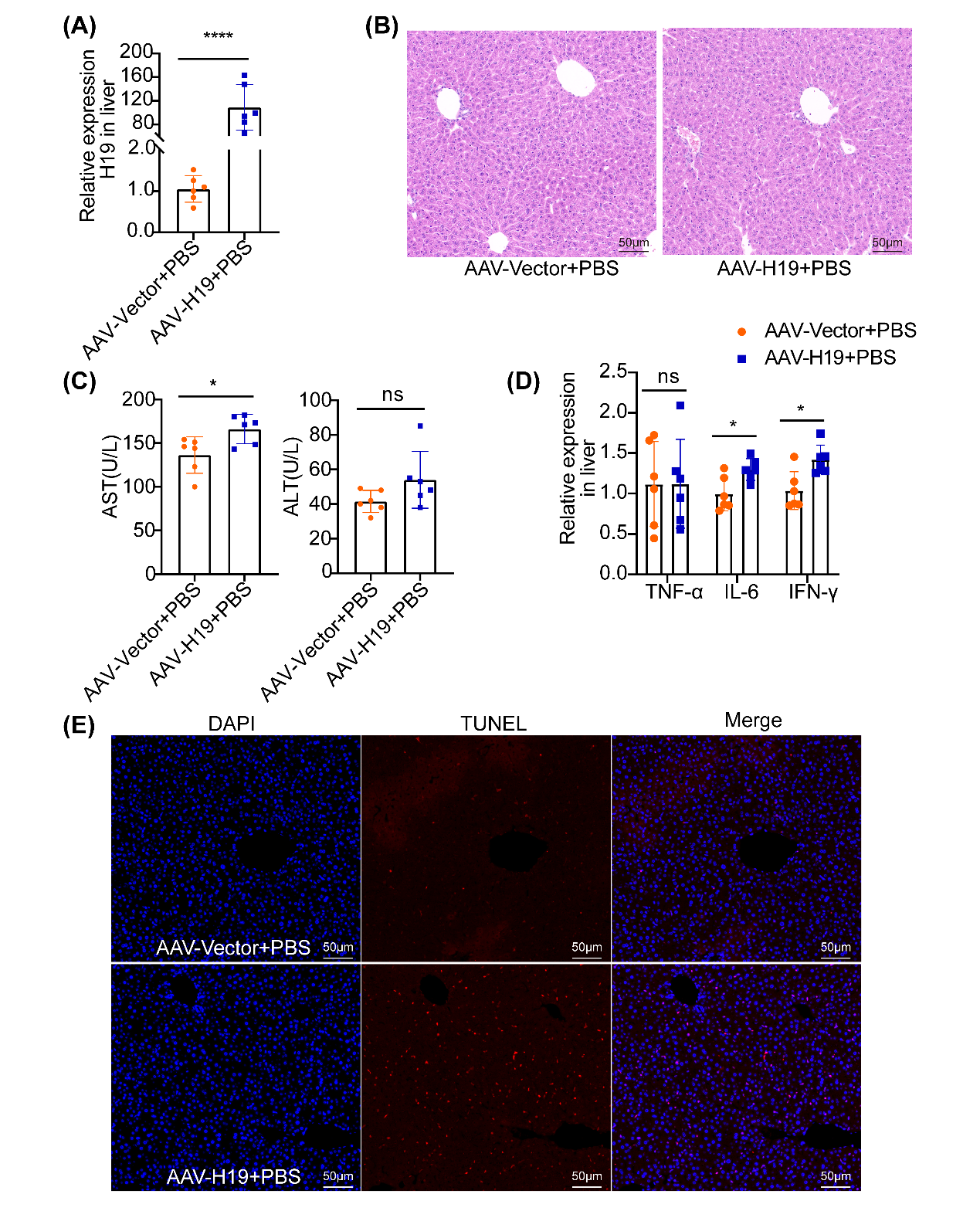


Figure S9. H19-overexpressed in the liver did not induce liver injury in mice treated with PBS. (A) RT-PCR showing the expression of H19 in the liver from PBS-treated mice (n=6). (B) Representative images showing hepatic injury, scale bar=50 μm. (C) The serum levels of ALT and AST in PBS-treated mice injected with either AAV-Vector or AAV-H19 (n=6). (D) RT-PCR showed the mRNA levels of inflammatory cytokines, including IL-6, TNF-α, and IFN-γ (n=6). (E) Representative images of TUNEL staining showing hepatocytes apoptosis in the liver, scale bar=50 μm. Statistical analysis showed the mean ±SD; ns: not statistically significant, *p<0.05, **p<0.01, ***p<0.001.


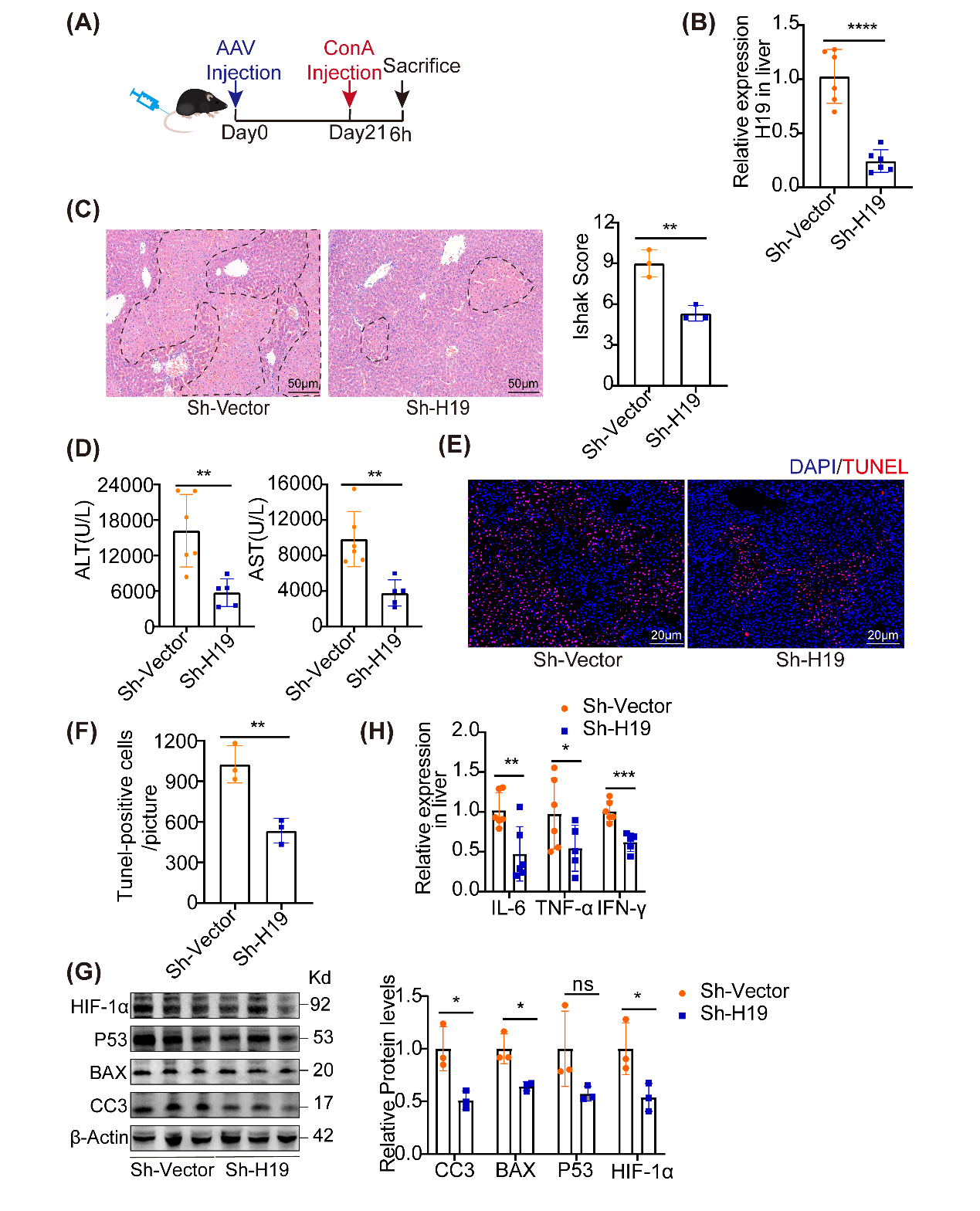


Figure S10. H19-knocked down attenuated ConA-induced hepatitis. (A) Mice were injected Sh-Vector/H19 via the tail vein before ConA treatment and were sacrificed. (B) RT-PCR showing the expression of H19 in the liver from ConA-treated mice (n=6). (C) Representative images showing hepatic injury and ISHAK scores (n=3), scale bar=50 μm (dashed line showed necrotic area). (D) The serum levels of ALT and AST in ConA-treated mice injected with either Sh-Vector or Sh-H19 (n=6). (E) Representative images of TUNEL staining showing hepatocytes apoptosis in the liver, scale bar=50 μm. (F) Statistical analysis of TUNEL-positive cells (n=3). (G) Western blot illustrating the expression of apoptotic proteins in the liver. (H) RT-PCR showed the mRNA levels of inflammatory cytokines, including IL-6, TNF-α, and IFN-γ (n=6). Statistical analysis showed the mean ±SD; ns: not statistically significant, *p<0.05, **p<0.01, ***p<0.001.


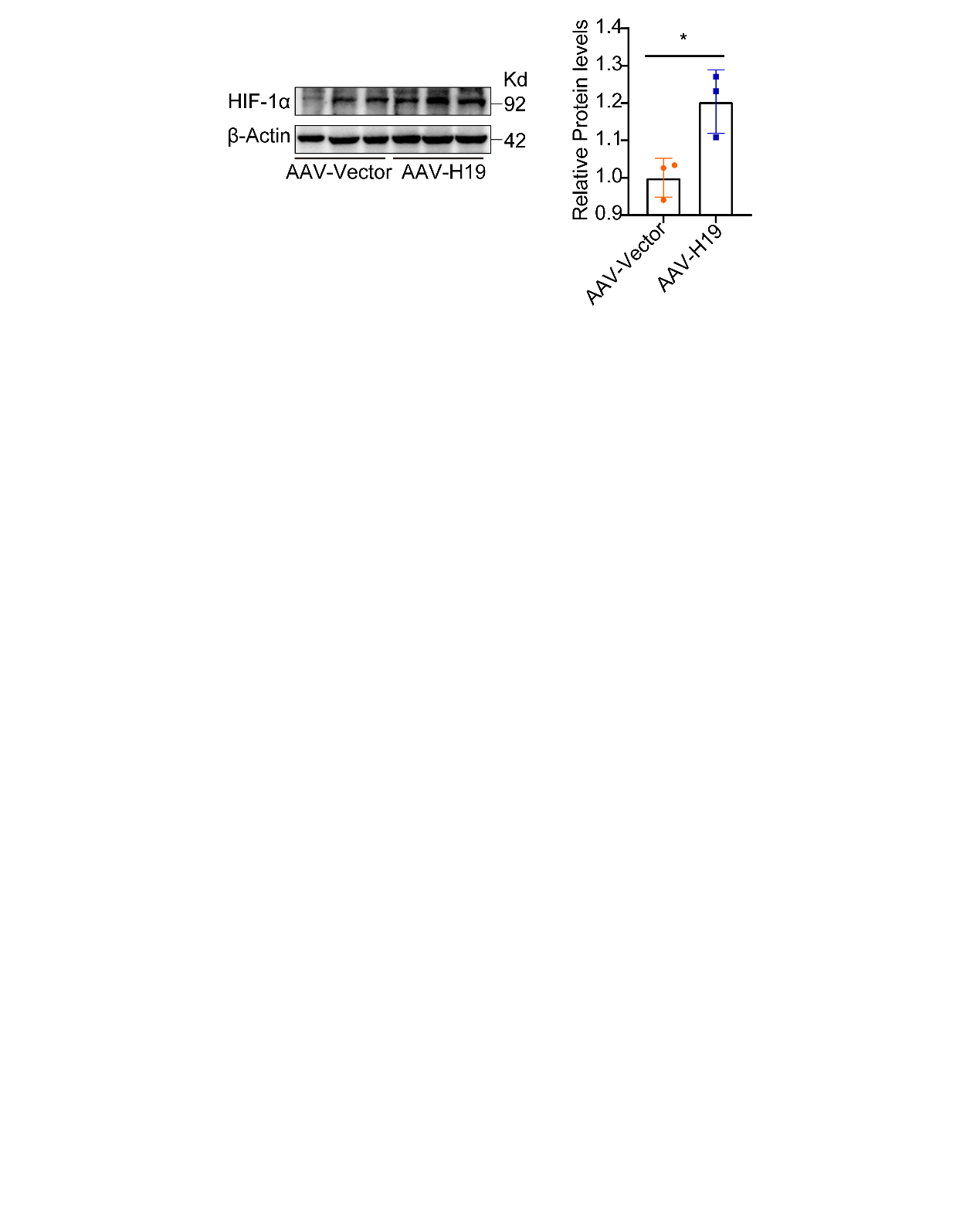


Figure S11. H19-overexpressed in the liver upregulated the level of HIF-1α, after ConA-induced liver injury.


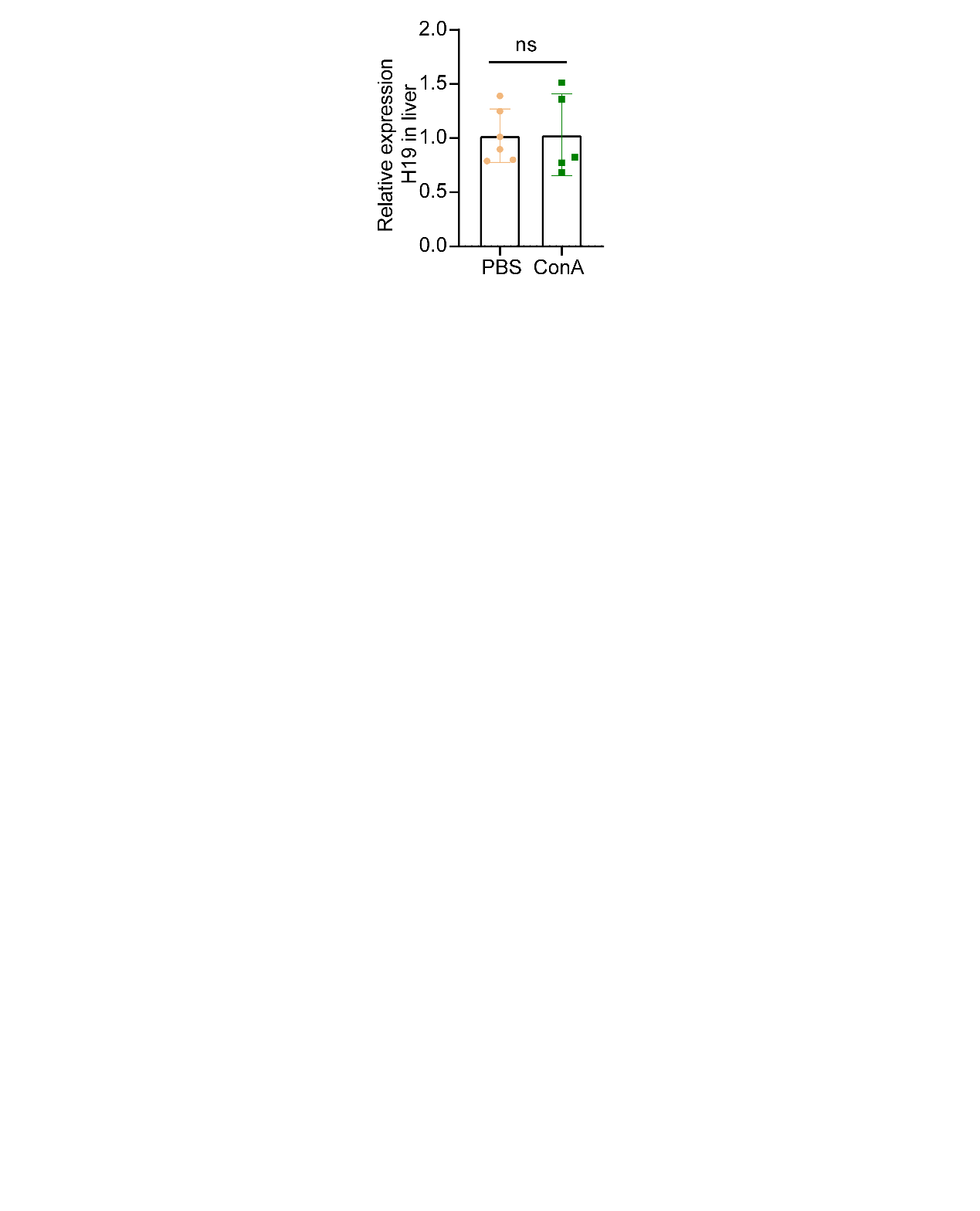


Figure S12. The expression of H19 in the liver of male mice, after ConA treatment.
